# Alzheimer’s disease: genetic background in the era of next-generation sequencing technologies

**DOI:** 10.1093/braincomms/fcag332

**Published:** 2026-09-16

**Authors:** Lydia Lukyova, Andrea Zatkova, Jaroslav Minarik, Zuzana Holesova, Jaroslav Budis, Marcel Kucharik, Vladimir Benes, Juraj Gazdarica, Tomas Szemes

**Affiliations:** Faculty of Natural Sciences, Department of Molecular Biology, Comenius University, Bratislava 841 04, Slovakia; Geneton Ltd., Bratislava 841 04, Slovakia; Genomics Core Facility at Comenius University Science Park, Bratislava 841 04, Slovakia; Geneton Ltd., Bratislava 841 04, Slovakia; Institute of Clinical and Translational Research, Biomedical Research Center of the Slovak Academy of Sciences, Bratislava 845 05, Slovakia; Faculty of Natural Sciences, Department of Molecular Biology, Comenius University, Bratislava 841 04, Slovakia; Institute of Clinical and Translational Research, Biomedical Research Center of the Slovak Academy of Sciences, Bratislava 845 05, Slovakia; Geneton Ltd., Bratislava 841 04, Slovakia; Geneton Ltd., Bratislava 841 04, Slovakia; Genomics Core Facility at Comenius University Science Park, Bratislava 841 04, Slovakia; Department of International Cooperation, Slovak Centre of Scientific and Technical Information, Bratislava 840 05, Slovakia; Geneton Ltd., Bratislava 841 04, Slovakia; Genomics Core Facility at Comenius University Science Park, Bratislava 841 04, Slovakia; Institute of Clinical and Translational Research, Biomedical Research Center of the Slovak Academy of Sciences, Bratislava 845 05, Slovakia; Scientific Core Facilities, Services & Technology Unit, European Molecular Biology Laboratory, Heidelberg D-69117, Germany; Geneton Ltd., Bratislava 841 04, Slovakia; Genomics Core Facility at Comenius University Science Park, Bratislava 841 04, Slovakia; Faculty of Natural Sciences, Department of Molecular Biology, Comenius University, Bratislava 841 04, Slovakia; Geneton Ltd., Bratislava 841 04, Slovakia; Genomics Core Facility at Comenius University Science Park, Bratislava 841 04, Slovakia

**Keywords:** Alzheimer’s disease, whole-genome sequencing, structural variants, somatic mosaicism, polygenic risk score

## Abstract

While rare, highly penetrant variants in *APP*, *PSEN1* and *PSEN2* are associated with early-onset Alzheimer’s disease, especially its familial form, the disease in general is shaped by a complex interplay of various genetic factors. Both common and rare variants across numerous loci, which were identified mainly during large genome-wide association studies, contribute to disease risk, phenotypic heterogeneity and progression in both early- and late-onset forms. This review synthesizes current understanding of the genetic architecture of Alzheimer's disease in the era of next-generation sequencing, with particular emphasis on variant classes beyond single nucleotide polymorphisms. Structural variants, including copy number variations, tandem repeat expansions and transposable element insertions, are considered as potential contributors to the ‘missing heritability’ in Alzheimer's disease. In addition, we discuss somatic mosaicism that represents another layer of its complexity. We highlight how whole-genome sequencing, combined with advanced bioinformatic pipelines and reference resources, enables systematic detection of different variant types. We also outline the translational potential of genetic discoveries, including polygenic risk and hazard score models for patient stratification, and underscore the importance of integrative genomic and multi-omic approaches in advancing precision medicine in Alzheimer's disease.

## Teaser

Lukyova *et al*. provide an overview of the genetic background of Alzheimer’s disease in the era of next-generation sequencing. By integrating knowledge on both small and structural variants, somatic mosaicism and polygenic risk/hazard modelling, they highlight emerging mechanisms that may explain missing heritability and highlight AI-driven multi-omic integration for personalized disease prediction.

## Graphical Abstract

**Graphical Abstract:**
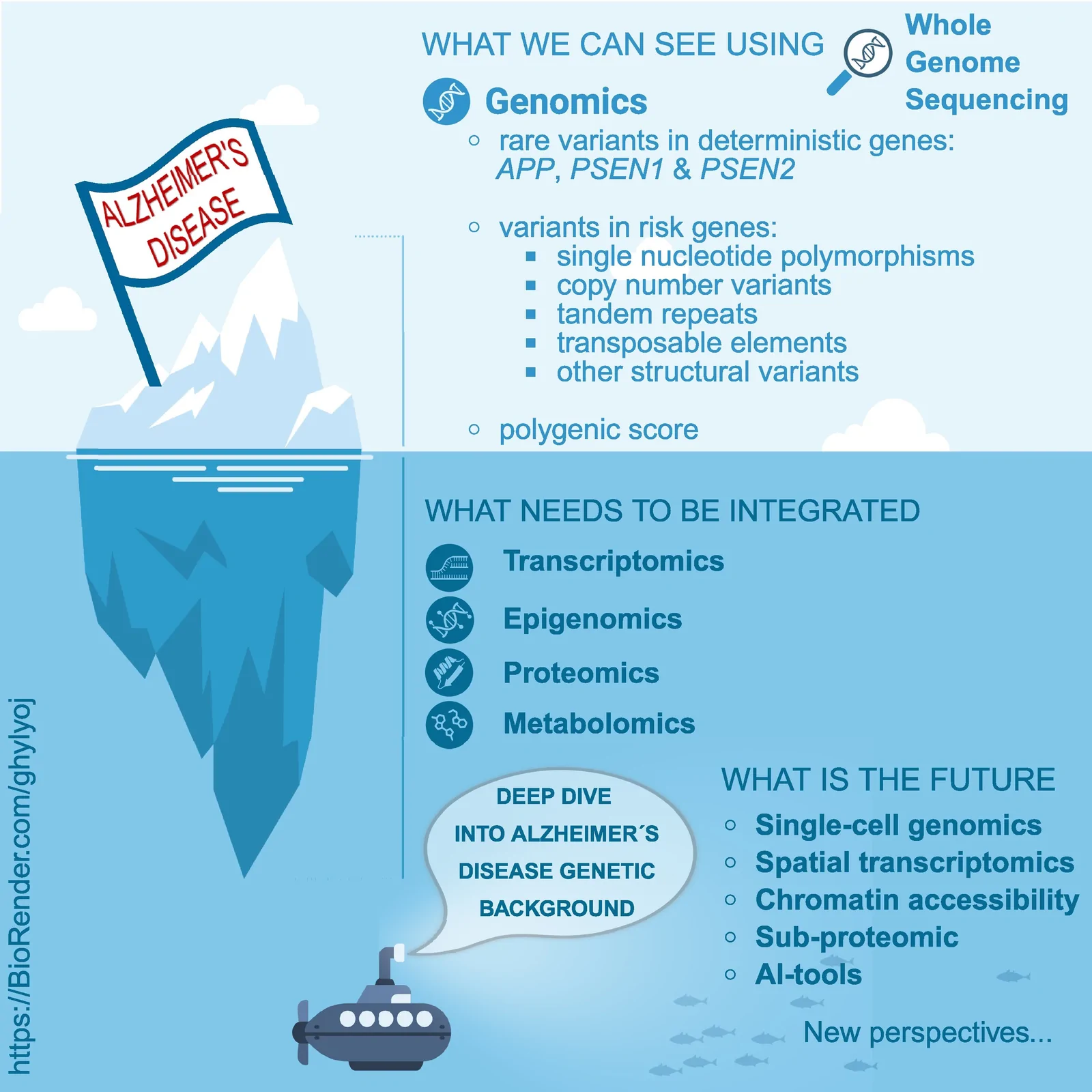

## Introduction

**Figure 1:**
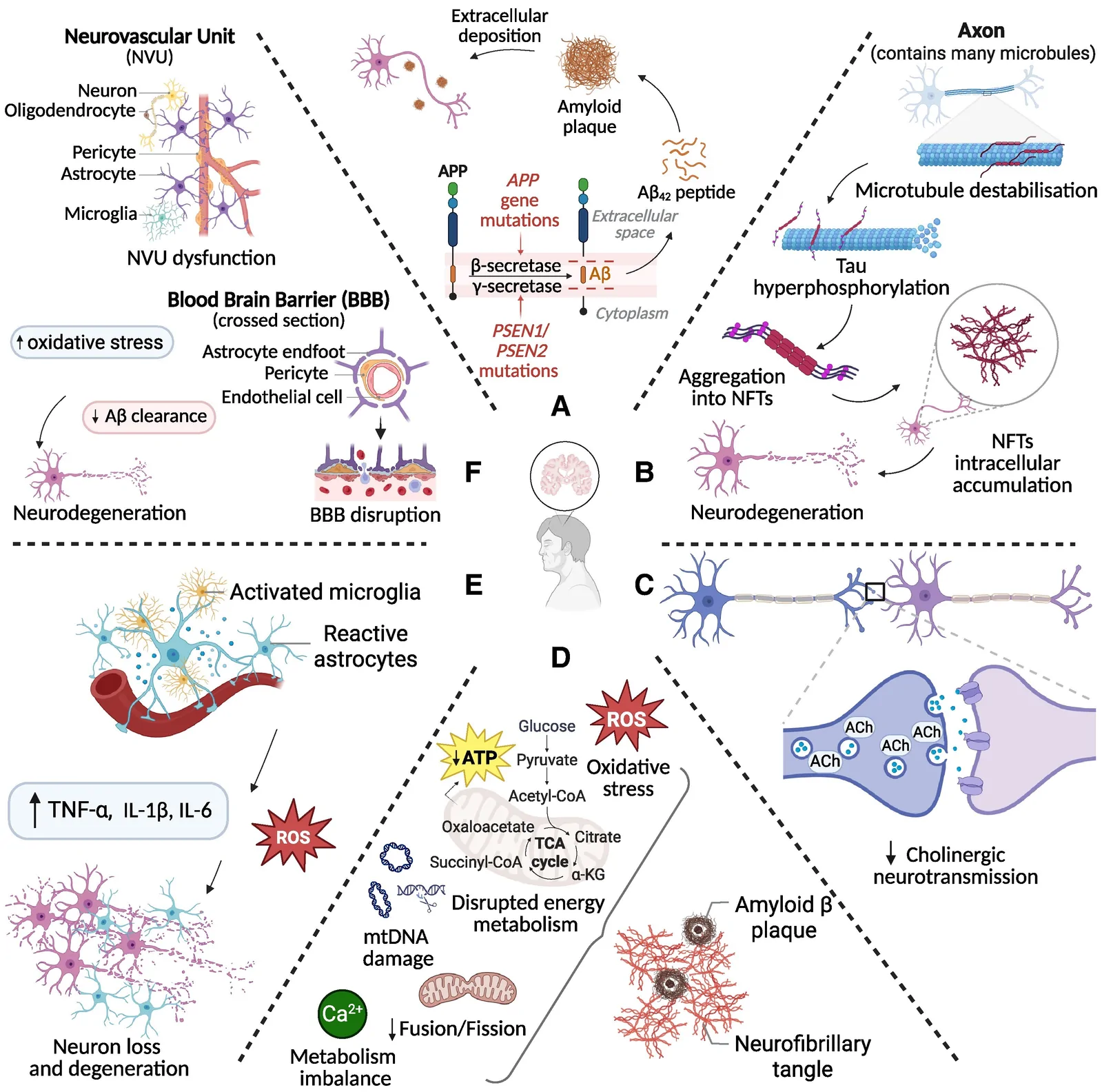
**The main hypotheses on the origin of Alzheimer’s disease and their underlying mechanisms (based on recent review** ^2^): **A**) Aβ hypothesis: Alzheimer's disease is initiated by an imbalance between the production and clearance of amyloid beta (Aβ) peptides, particularly the aggregation-prone Aβ_42_ isoform that arises from amyloid precursor protein (APP) processing via the amyloidogenic pathway, which involves β- and γ-secretases. These peptides aggregate into soluble oligomers and insoluble fibrils that deposit as plaques, disrupting synaptic function and triggering neuroinflammation. (**B**) The tau hypothesis: Abnormal hyperphosphorylation of the tau protein causes it to detach from microtubules and form aggregated neurofibrillary tangles (NFTs), the build-up of which leads to neuronal death. (**C**) Cholinergic hypothesis: Cognitive decline in Alzheimer's disease is primarily due to a deficit in the neurotransmitter acetylcholine, resulting from the degeneration of cholinergic neurons. (**D**) Mitochondrial stress hypothesis: Mitochondrial dysfunction contributes to the formation of NFTs and senile plaques. This process is mediated by a cascade of cellular disturbances, including increased production of reactive oxygen species (ROS), increased oxidative stress, impaired energy metabolism and dysregulated calcium homeostasis. (**E**) Inflammation hypothesis: Prolonged activation of the brain's innate immune system, which primarily involves microglia and astrocytes, is a driver of Alzheimer's disease progression. The presence of aggregated Aβ in neural tissue promotes the activation of these glial cells, leading to increased production of proinflammatory cytokines (TNF-α, IL-1β and IL-6) as well as ROS. (**F**) Neurovascular hypothesis: Impaired cerebral blood flow and the disruption of the neurovascular unit (NVU) lead to chronic hypoperfusion, which in turn reduces Aβ clearance, promotes oxidative stress and initiates inflammation. TNF, tumour necrosis factor; IL, interleukin; mtDNA, mitochondrial DNA; Ach, acetylcholine (created in BioRender. Pös, O. (2026) https://BioRender.com/qd3g56m).

Alzheimer's disease is a progressive neurodegenerative disease characterized by a specific pattern of cognitive and functional decline that correlates with advancing age and is associated with distinct neuropathological changes.^1^ It is the most prevalent type of dementia, accounting for 60–70% of cases. There are various hypotheses describing the possible pathophysiology of Alzheimer's disease (Fig. 1), of which the main ones are the amyloid beta (Aβ) and tau hypotheses (Fig. 1A and B), supported by the most extensive body of experimental and clinical data. In general, Alzheimer's disease is defined by the extracellular deposition of senile plaques, primarily composed of Aβ peptides, and the intracellular accumulation of neurofibrillary tangles (NFTs) formed by abnormally hyperphosphorylated tau protein,^3^ both of which are central to the process of neurodegeneration and cognitive decline.^4^ Tau protein is a microtubule-associated protein crucial for stabilizing neuronal microtubules and enabling axonal transport,^5^ while Aβ is a naturally occurring protein by-product that plays several physiological roles in the brain at lower, soluble levels, including antimicrobial protection, sealing blood-brain barrier leaks, promoting recovery from brain injury, supporting synapse formation and regulating long-term potentiation (memory consolidation) recently reviewed.^6^ More details on Alzheimer's disease pathophysiology mechanisms can be found in a recent review.^2^

Based on the age of onset, Alzheimer's disease can be classified as early-onset Alzheimer's disease (EOAD, before age 65) or late-onset Alzheimer's disease (LOAD, begins after age 65). Many metabolic and lifestyle factors are linked to Alzheimer's disease risk, but genetics contributes significantly to its complex pathophysiology, with the heritability estimates based on twin studies ranging from 60% to 80%, especially for LOAD.^7,8^ Lower values have been obtained in analyses of individual-level genome-wide association studies (GWAS) data from unrelated individuals (from 24% to 55%).^9^

EOAD is often linked to familial Alzheimer's disease (fAD), an autosomal dominant form often caused by variants in or near the Aβ region of the *APP* gene,^10^ or *PSEN1* and *PSEN2* variants, which alter γ-secretase function (Fig. 1A), increasing the production of aggregation-prone Aβ_42_ and causing midlife Aβ deposition.^4^ While all autosomal dominant fAD cases are EOAD, not all EOAD cases are familial; many are sporadic with unclear genetic and environmental causes;^11^ therefore, only a minority (1–2%) of EOAD cases can be explained solely by known rare variants (in the *APP*, *PSEN1*, or *PSEN2* gene); rather, most involve common genetic variants in other Alzheimer's disease associated genes.

LOAD is more common and often referred to as sporadic Alzheimer's disease, though familial clustering is observed in some cases. LOAD is influenced by complex genetic, environmental and lifestyle factors. Genetic contributors are mainly common but incompletely penetrant variants in numerous genes, such as those in the *APOE* gene.^3^ Many of these variants have been discovered thanks to Alzheimer's disease genome-wide association studies (AD-GWAS) that rather than focusing on single candidate genes scan hundreds of thousands to millions of small nucleotide polymorphisms (SNPs) in large populations to uncover loci linked to the disease. Thus, most cases of LOAD, but many EOAD cases as well, appear to be influenced by numerous genetic variants, each contributing a small effect. Consequently, Alzheimer's disease is often regarded as a complex polygenic disorder^12^ shaped by both genetics and the environment.

**Figure 2:**
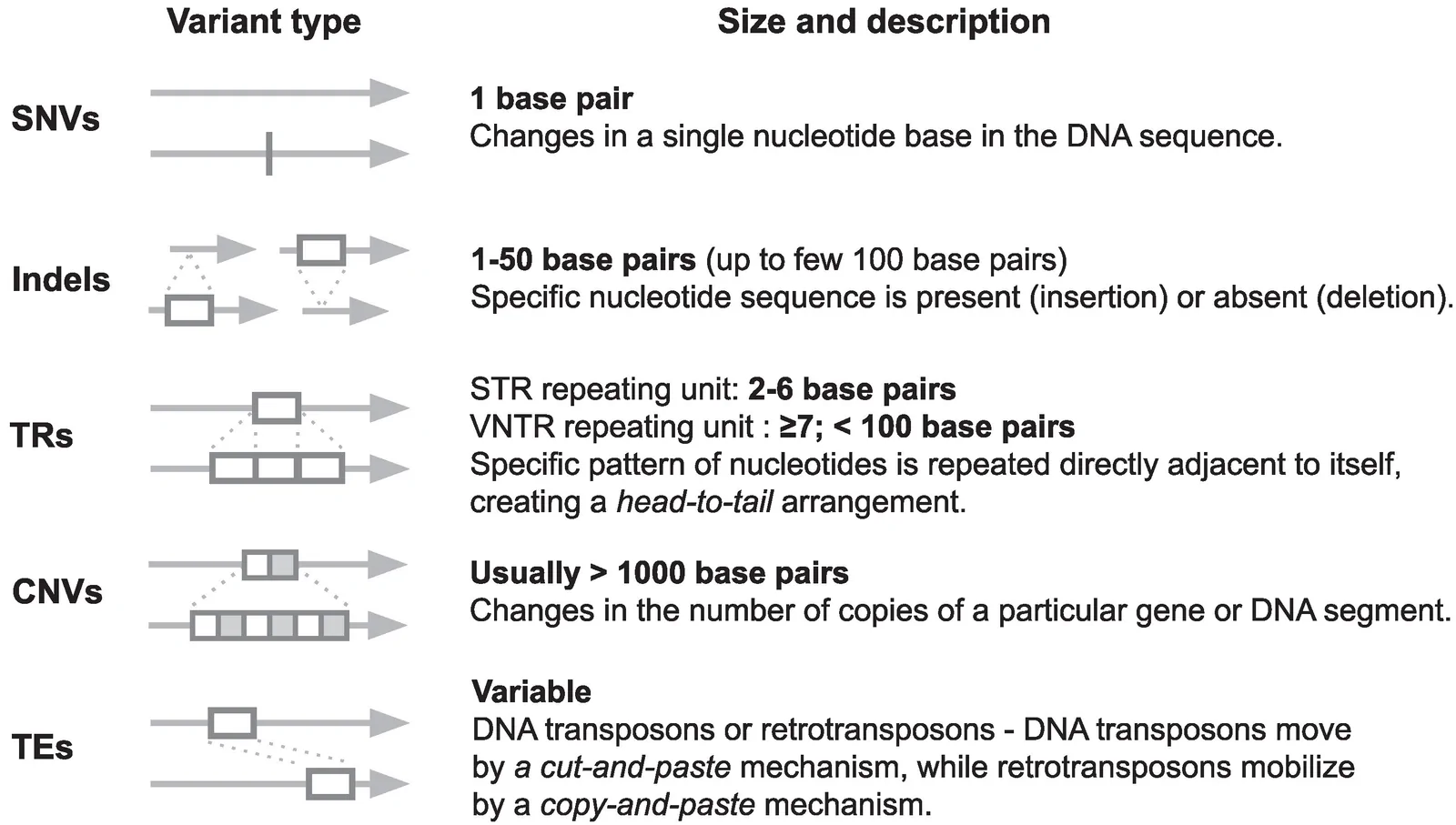
**Simplified representation and main characteristics of the different genetic variant types discussed in this review.** Single-nucleotide variants (SNVs) represent the smallest genomic changes and can impact gene regulation or protein function. By definition, single nucleotide polymorphisms (SNPs) are SNVs with a minor allele frequency of ≥1%. Additionally, small insertions and deletions (indels) may disrupt coding sequences or influence splicing and are readily identified by variant callers during point/small variants analysis from short-read WGS data. Copy number variations (CNVs) are larger structural variants that alter gene dosage (gain/loss) and have been linked to neurodevelopmental and neurodegenerative conditions. Tandem repeats (TRs), including short tandem repeats (STRs) and variable number tandem repeats (VNTRs), usually influence gene expression and are implicated in repeat expansion disorders with neurological manifestations. Transposable elements (TEs), such as Alu, LINE-1 and SVA elements, can disrupt genes or regulatory regions through insertional mutagenesis and are increasingly recognized for their role in genome instability and somatic mosaicism in the ageing brain and Alzheimer’s disease. CNVs, TEs and TRs identification from short-read WGS or long-read sequencing data require dedicated tools, pipelines and databases that are discussed in the text (created by Google Slides).

Data on genetic variants and their potential role in Alzheimer's disease pathophysiology is growing with the use of next-generation sequencing (NGS) technologies, especially whole-genome sequencing (WGS), which is used also in large-scale population studies and holds strong potential for widespread clinical adoption. In this review, we summarize the current understanding of the complex roles played by different types of genetic variants in Alzheimer's disease (Fig. 2). While early studies focused mainly on common single nucleotide polymorphisms (SNPs), these explain only a fraction of the genetic risk. Therefore, we discuss not only small variants (including SNPs identified in AD-GWAS studies) but also larger-scale changes or structural variants (SVs) that can contribute to Alzheimer's disease risk^13^ and possibly help to explain so-called ‘missing heritability’.^14^ These include copy number variations (CNVs), transposable elements (TEs)-related changes and tandem repeats (TRs). Selected examples of different variant types subdivided by the functional category of involved Alzheimer's disease genes are summarized in Supplementary Table 2. We also examine another critical layer of genetic complexity in Alzheimer's disease: somatic mosaicism in brain tissues, a phenomenon in which postzygotic *de novo* variants create genetically distinct cell populations within a single individual.^15^ Moreover, we discuss the potential of polygenic risk score (PGS) and polygenic hazard score (PHS) calculations in Alzheimer's disease risk assessment, along with the integration of genomic data with other *omics* and AI-driven analyses to enhance biomarker discovery and individualized risk prediction. Thus, WGS enables new approaches that reveal layers of Alzheimer's disease heritability beyond traditional SNP/GWAS models and are reshaping the future of risk stratification.

## Potential of WGS technologies for Alzheimer's disease research and risk stratification

While whole exome sequencing (WES) can be used to identify variants within the coding region of the major Alzheimer’s disease determining or influencing genes, here, we focus specifically on the use of WGS that provides the most comprehensive view of an individual's genetic makeup. Unlike more targeted methods, in a single analysis, WGS covers the entire genome, including both coding and non-coding regions, and enables the detection of a wider variety of genetic variants. This is particularly valuable for research and diagnosis of complex diseases, where the genetic cause(s) may reside outside of the protein-coding exome. Depending on read length and WGS strategy, WGS data can be generated using short-read (such as Illumina) and long-read platforms (such as PacBio HiFi, Oxford Nanopore (ONT)), with typical read length from 25 to 400 bp and from thousands to millions of bp, respectively.^16^ Owing to its higher accuracy and throughput, short-read sequencing is widely used for detecting single-nucleotide variants (SNVs) and small indels; however, it has some limitations in reliably detecting variants in repetitive regions (including TR expansions) and larger SVs, which are usually better detected by long-reads. A more detailed overview of these technologies is provided in another review article.^17^

Despite the still relatively high cost and the robust computational and data storage infrastructure needed for clinical application, the WGS offers significant opportunities, offering a single, comprehensive approach, replacing the need for multiple separate tests. It has already been reported as an effective tool for the diagnosis of neurological disorders because of its strong specificity and sensitivity,^18^ indicating its clinical potential also for complex diseases. Once WGS becomes routine in healthcare, it is likely that this genetic information will become a standard component of many individuals’ medical records. With continually improving analytical tools and databases, WGS data can be reanalysed whenever necessary to obtain an updated view of a patient's condition and an improved understanding of disease risk or diagnosis. Within the specific context of Alzheimer’s disease, WGS has recently been used in the Alzheimer's disease sequencing project as a tool for better understanding the complexity and genetic background of the disease, aiming at detecting missing genetic factors influencing Alzheimer's disease.^19^ Nevertheless, full interpretation of WGS data requires integration with other *omics* layers, as discussed later in this review.

## Rare and common small-scale variants in Alzheimer's disease

Small-scale DNA variants, such as SNVs (which refer to any single-nucleotide variant), SNPs (common SNVs with minor allele frequency ≥ 1%) as well as small indels play a key role in understanding the genetic architecture of Alzheimer's disease. These variants and their specific roles in the disease have recently been reviewed in more detail.^20^ They are mainly rare high-impact variants in genes like *APP*, *PSEN1* and *PSEN2* that can explain some fAD/EOAD cases (Table 1). It is important to emphasize that rare pathogenic variants in *MAPT* gene encoding tau protein are not among them, but *MAPT* variants are instead firmly established as the cause of ‘tauopathies’ (diseases characterized by tau tangles, like familial frontotemporal dementia (FTD)).^5^ However, certain common variants within *MAPT* have been identified as Alzheimer's disease risk modifiers (such as p.Ala152Thr), which are not fully penetrant and act by promoting tau protein aggregation.^21^

**Table 1:** Key genes and SNPs associated with Alzheimer's disease risk

Gene	Genome location	Key functions	The most studied variants	Association with Alzheimer's disease
*APP*	21q21.3	Precursor for Aβ; cleaved by β/γ-secretase	118 variants, pathogenic (e.g. V717I ‘London’, KM670/671NL ‘Swedish’)	Strongly linked to fAD; many mutations increase Aβ_42_/Aβ_40_ ratio and support tau pathology
*PSEN1*	14q24.3	Part of γ-secretase; Aβ production	382 variants; mostly missense	Most severe fAD cases; often de novo
*PSEN2*	1q31-q42	Homologous to PSEN1; γ-secretase component	92 variants, nonsynonymous substitutions and frameshift mutations	Rare; milder fAD/EOAD (onset ∼40+ years); increased Aβ_42_/Aβ_40_ ratio
*APOE*	19q13.32	Lipid transport; binds Aβ	ɛ3 (neutral), ɛ4 (risk allele); rs429358 & rs7412 define isoforms	Major LOAD risk factor; ɛ4 increases risk 2–3× (1 copy), up to 12× (2 copies)
*TOMM40*	19q13.32	Mitochondrial protein import	rs157581, rs11556505	Modulates Alzheimer's disease risk with APOE; affects mitochondrial function
*TREM2*	6p21	Microglial activation; Aβ clearance	R47H (rs75932628), R62H, T96K	Heterozygous → LOAD/FTD risk; homozygous → early-onset dementia (e.g. Nasu-Hakola)
*CD33*	19q13.33	Microglial receptor; Aβ clearance inhibition	rs3865444 (C allele = risk), rs3826656	Increased expression—reduced clearance of Aβ and cognitive decline
*CR1*	1q32	Complement activation; Aβ clearance	rs6656401, rs4844609, rs3818361	Related to Alzheimer's disease risk and late-life cognitive decline
*CLU*	8p21-p12	Lipid transport; binds Aβ; immune modulation	rs11136000 (C allele = risk)	3rd top LOAD risk gene; involved in stress and cell survival pathways
*BIN1*	2q14–q21	Glutamatergic neuronal activity, tau pathology	rs744373, rs6733839, rs7561528	2nd strongest LOAD risk gene; links mostly to tau, not Aβ
*PICALM*	11q14	Endocytosis, APP trafficking, autophagy	rs3851179 (G allele = risk)	Modulates Aβ production, lysosomal function, tau pathology
*ABCA7*	19p13.3	Lipid metabolism; Aβ phagocytosis	rs3764650 (G allele = risk)	Impacts both EOAD and LOAD risk; regulates cholesterol and APP processing
*CD2AP*	6p12.3	Cytoskeletal structure; APP transport	rs9349407 (LOAD), rs9473117 (EOAD)	Affects Aβ degradation; important for cytoskeletal integrity
*MS4A* gene cluster (e.g *MS4A4A* and *MS4A6E*)	11q12	Microglial expression; TREM2 regulation	rs6591561 (risk)	Alters CSF sTREM2 levels; influences immune response in Alzheimer's disease
*SORL1*	11q24.1	Regulates APP transport and Aβ production	Over 500 different variants (e.g. rs117260922)	Associated with both EOAD and LOAD; reduced expression leads to increased Aβ production

The number of variants and rs numbers are from available gene specific databases (accessed April 2026). EOAD, early-onset Alzheimer’s disease; fAD, familial Alzheimer’s disease; FT, familial frontotemporal dementia; LOAD, late-onset Alzheimer’s disease; rs, reference SNP cluster identifier (also called rsID) for SNP variants.

However, the majority of Alzheimer's disease cases are not monogenic. Most LOAD (but also EOAD) cases are influenced by common SNPs in many other genes that modulate its risk (Table 1). Many of them have been identified through GWAS studies,^8^ which have become a cornerstone of Alzheimer's disease research by enabling the systematic identification of common genetic variants associated with disease risk across the entire genome. Importantly, GWAS findings have shifted the understanding of Alzheimer's disease from a purely amyloid-centric disorder to a more complex, multifactorial condition. The major and best-established genetic risk locus for Alzheimer's disease is *APOE*. The three primary *APOE* isoforms ɛ4, ɛ3 and ɛ2 are determined by the specific combination of two SNPs (rs429358 and rs7412) located within the gene.^22^ Of these, *APOE* ɛ4 (rs429358-C and rs7412-C) significantly increases the risk of LOAD, most likely through the impact on myelination.^23^ The importance of this risk factor is underpinned by the fact that approximately 40% of patients diagnosed with Alzheimer's disease carry at least one copy of the *APOE* ɛ4.^24^

Thanks to the use of large biobanks for GWAS, the identification of other SNPs and genetic loci linked to Alzheimer's disease has recently accelerated and some new discoveries are still to be expected. In the past decade alone, the number of such loci has increased from 20 to over 118.^14,25^ For example, common (but also rare) risk-associated SNPs were identified in genes such as *CLU*, *BIN1*, *PICALM* and *TREM2* (Table 1), involved in pathways like amyloid processing, lipid metabolism, inflammation and synaptic function.^26^ Individually, these SNPs usually confer modest effects, but together they contribute to a polygenic risk profile that influences susceptibility, age of onset and disease progression, making them valuable for research into mechanisms, risk prediction and potential therapeutic targets.^8^

### Rare variant burden

Interestingly, sequencing in fAD pedigrees negative for *APP, PSEN1* or *PSEN2* variants together with subsequent larger exome studies indicates that truncating or ultra-rare missense variants (with a dominant negative effect) in different domains of the SORL1 protein have a disease-causing effect in Alzheimer's disease.^27,28^ Therefore, further discoveries are still expected in this field, especially thanks to rare variant burden analyses that compare the prevalence of rare, potentially deleterious variants within a particular gene in individuals with Alzheimer's disease to unaffected controls. Since individual rare variants are typically observed in only a small number of individuals, analyses of single variants often lack sufficient statistical power. By aggregating multiple variants within a gene, such burden tests enhance the ability to identify gene-level associations that might otherwise remain undetected. Such variants have effect sizes larger than GWAS loci (moderate to large effect size), and it is believed they could elucidate part of the missing heritability in Alzheimer's disease. This approach gained significant traction especially following the discovery of rare variants in *TREM2*^29^ and loss-of-function variants in *ABCA7.*^30^ So far, at least 13 genes have been reported that either increase or decrease the Alzheimer’s disease risk through such rare genetic variants, and other promising candidate genes with a rare variant burden signal have been identified.^31^

### Small-scale DNA variant genotyping and interpretation

Genotyping SNVs and small indels depends on the data type, scale and accuracy needs. Identification of rare high-impact variants linked to EOAD/fAD (in genes *APP*, *PSEN1* and *PSEN2,* Table 1) and evaluation of their pathogenicity follow the same rules as for monogenic diseases (American College of Medical Genetics and Genomics (ACMG) criteria^32^), using databases such as gnomAD,^33,34^ ClinVar,^35^ and OMIM^36^ can aid interpretation.

GWASs have traditionally relied on DNA arrays to analyse known SNPs via hybridization, but this method misses novel variants. New solutions have been developed to extend coverage, such as SNP imputation (uses reference panels such as the 1000 Genomes Project) and more recent GWASs already use WGS technology to analyse SNPs genome-wide. GWAS typically identifies variants with small effect sizes, usually requiring very large sample sizes, and translating these associations into biological mechanisms remains a major ongoing challenge. Bias towards European ancestry may be reduced by including more individuals from different populations.^37^ Details on GWAS methodology can be found in a recent review.^38^

Genotyping arrays followed by genotype imputation can also be used to increase genomic coverage in general and improve power for locus discovery. This approach has enabled the identification of numerous Alzheimer's disease risk loci, including genes involved in amyloid processing, lipid metabolism and immune pathways. However, the advantage of WGS approach is that it provides simultaneous genotyping (for GWAS) and identification of possible rare ‘determining’ variants.^39^

Based on the sequencing technology and laboratory, several tools can be used that employ statistical or machine learning (ML) models to distinguish true variants from erroneous calls, which are typically caused by low coverage, mapping ambiguity or other technical biases. To test genetic associations of identified SNPs with continuous or binary traits/phenotypes, linear or logistic regression models are used, respectively, applying a Bonferroni significance threshold (*P* < 5 × 10^−8^) to reduce false positives. Common approaches for rare variant burden testing include Combined Multivariate and Collapsing (CMC),^40^ Sequence Kernel Association Test (SKAT)^41^ and SKAT-O.^42^

## Copy number variation variants (CNVs)

CNVs are deletions or duplications of genomic segments (typically ranging from kilobases to megabases) that alter gene dosage and contribute substantially to human genetic diversity and disease susceptibility.^43^ In Alzheimer’s disease, CNVs represent an important class of structural variation because they can affect both canonical Alzheimer’s disease genes and broader pathways related to immunity, lipid metabolism, synaptic function and neuronal resilience.

### Biological relevance of CNVs in Alzheimer's disease

Among the best-established CNVs in Alzheimer's disease is *APP* duplication, which causes autosomal dominant EOAD by increasing amyloid precursor protein (APP) dosage and accelerating Aβ accumulation.^44^ Deletions affecting *PSEN1* exon 9 have likewise been linked to rare fAD cases.^45^

Beyond these canonical loci, CNVs in immune-related genes such as *CR1* and *CHRFAM7A* have also been implicated in Alzheimer’s disease risk. Duplication of the low-copy repeat within the *CR1* locus has been associated with increased risk, likely through altered complement activity,^46,47^ whereas variation involving *CHRFAM7A* may affect α7 nicotinic acetylcholine receptor signalling.^46,48^

Recent exome-wide analyses have highlighted additional rare CNVs in Alzheimer’s disease, including events affecting genes such as *MAPT*, *ABCA1*, *ABCA7*, *TYROBP* and *CTSB*, as well as recurrent CNVs in regions such as 22q11.21.^49^ Other reported CNVs involve loci linked to synaptic and neurodevelopmental function, detoxification (*SULT1A3/4*) and carbohydrate metabolism (*AMY1A*),^50-53^ with additional network-based analyses suggesting broader effects on pathways related to inflammation, signalling and disease progression.^54^ Together, these findings suggest that CNVs may influence Alzheimer’s disease susceptibility, age of onset and clinical heterogeneity through dosage effects across multiple disease-relevant pathways. Selected CNVs associated with Alzheimer’s disease, including recurrent pathogenic events, candidate loci and variants linked to clinical heterogeneity, are summarized in Supplementary Table 1.

One important mechanism by which CNVs may contribute to Alzheimer’s disease is altered gene expression. Integrative genomic and transcriptomic studies have shown that CNVs can modulate genes involved in memory, immunity and vascular function, including *CREB1*, *FAM119A*, *HLA-DRA*, *TLR1*, *PLAU* and *PLGRKT.*^55,56^ An example is an 8-kb deletion upstream of *CREB1*, which increases Alzheimer’s disease risk by disrupting regulation within a linked chromosomal region.^55^ CNVs may also influence chromatin organization and transcriptional regulation in neurons.^57^ These effects may be relevant not only for disease risk, but also for phenotype modulation, including psychosis, progression-related traits and other aspects of clinical variability in Alzheimer’s disease.^51,54,58^

### Detection of CNVs in the NGS era

CNV analysis from an NGS perspective remains challenging because CNVs must be inferred indirectly from sequencing signals such as read depth, read-pair orientation, split reads and local assembly, which often generate large and noisy callsets.^59^ This is consistent with large Alzheimer’s disease WGS studies, in which CNV or SV detection relied on multiple callers and subsequent harmonization or joint genotyping before downstream analysis.^51,52,60^ Short-read WGS generally performs well for larger CNVs and genome-wide dosage profiling, but it is less reliable in repetitive regions and often provides limited breakpoint resolution for complex events. Long-read sequencing can overcome part of these limitations by improving breakpoint definition, resolving repetitive or structurally complex loci and facilitating phasing, although its current use in Alzheimer’s disease remains more limited because of cost, throughput and analytical demands.^61^ WGS can improve resolution of rare or complex CNVs compared with array-based approaches, but robust analysis still requires stringent filtering and validation.^51,52,60^ CNVs are then typically interpreted by annotating gene content, overlap with known Alzheimer’s disease loci and predicted dosage sensitivity, followed by integration with diagnosis, age at onset, Braak and CERAD scores for quantifying neuropathological features of Alzheimer’s disease, biomarker profiles and transcriptomic data.^51,52^ Large Alzheimer’s disease multi-omics studies have further used CNV-gene-trait correlation networks to link CNVs with gene-expression changes and disease-relevant pathways.^51^ For germline CNVs, ACMG/ClinGen-based frameworks and tools such as ClassifyCNV can support classification.^62^ For somatic or single-cell CNVs, interpretation requires particularly stringent filtering, denoising, and orthogonal validation because low-frequency events are highly susceptible to technical artefacts.^63^ This caution is also supported by single-cell studies in Alzheimer’s disease neurons showing that conclusions depend strongly on post-calling filtering and noise reduction.^63^

## Transposable element-related variants (TEs)

TEs, including long interspersed nuclear elements (LINEs), short interspersed nuclear elements (SINEs), LTR retrotransposons and DNA transposons, comprise a substantial fraction of the human genome.^64^ Although most are epigenetically silenced, a subset remains active and can promote genomic instability through insertions, deletions and rearrangements.^65^ In Alzheimer’s disease, TEs are increasingly considered a potential source of both genomic and regulatory variation.

### TE-related variants and their relevance in Alzheimer’s disease

Among human TEs, SINE-VNTR-Alu (SVA) elements are a hominid-specific class of non-autonomous retrotransposons that may contribute to Alzheimer's disease-relevant repeat variation.^66^ In Alzheimer's disease-associated loci, structural variation within SVA elements, including variants near *BIN1*, has been linked to gene-regulatory effects, and STR expansions enriched within SVA retrotransposons have also been reported in Alzheimer’s disease.^66,67^ Alu insertions within *TOMM40* intron 6, a region in strong linkage disequilibrium with *APOE*, have also been proposed to contribute to Alzheimer's disease-related genetic variation.^68^ The poly-T repeat rs10524523 co-segregates with *APOE* isoforms, with longer alleles associated with *APOE* ɛ4 and shorter variants with ɛ2/ɛ3.^68^ An Alu-associated structural variant in the *TMEM106B* 3′UTR has also been linked to dementia-related traits, further supporting the view that TE-derived non-coding variation may modify disease-relevant phenotypes beyond canonical Alzheimer’s disease risk loci.^60^ Collectively, these observations suggest that TE-derived variants may modulate Alzheimer’s disease susceptibility through both regulatory effects and linkage with established risk loci.

TEs may also contribute to disease through transcriptional dysregulation. A TE expression quantitative trait loci analysis identified more than 26 000 TE loci associated with gene expression in aged Alzheimer’s disease brains, including a SINE element linked to repression of *C1QTNF4.*^69^ Tau pathology has likewise been associated with activation of retrotransposons, including LINE-1 and endogenous retroviruses, in post-mortem human brains and experimental models.^70^

### Detection of TE-variants in the NGS era

TEs represent one of the most technically challenging classes of variants to analyse using NGS, owing to their repetitive sequence content and frequent occurrence in structurally complex loci. Such sequences are poorly resolved by standard short-read approaches, which may miss insertions, misclassify breakpoints, or yield ambiguous alignments. Long-read platforms such as PacBio and ONT improve the detection of full-length insertions and structurally complex TE loci. However, reliable analysis still depends on specialized bioinformatic workflows, stringent filtering and careful downstream interpretation, particularly when linking TE-derived calls to Alzheimer's disease-relevant genes or regulatory mechanisms.^71,72^

## Tandem repeat variants (TRs) in Alzheimer’s disease

TRs, comprising 3–6% of the genome and found at more than 2 million loci, may represent another key source of genetic variation in Alzheimer’s disease.^73^ TRs generally vary by motif size and structure: short tandem repeats (STRs) (2–6 bp) and VNTRs (≥7 bp), with some VNTRs spanning full genes.^74^ While not all TRs are variable, if they have multiple alleles, they potentially exert greater functional effects than do biallelic SNPs. Owing to their high degree of polymorphism and instability, STRs are implicated in more than 40 monogenic neurodegenerative disorders, in which disease severity often correlates with expansion size (for review see^75^). TR changes can cause disease through mechanisms depending on the repeat location, sequence composition and gene context. Many known diseases causing STRs lie within exons, introns, or UTRs, but their impact on protein functions goes beyond direct coding roles (reviewed recently^73^), and many are known to colocalize with enhancers or transcription start sites, affecting gene regulation.^76^ The majority of STRs, however, are intergenic, and their changes can modulate gene expression or chromatin organization, often in a tissue-specific manner.^77^ Recent studies have also linked an increased burden of STR expansions to polygenic disorders such as autism and schizophrenia,^78-80^ confirming a potential role for TRs in Alzheimer’s disease risk.

### TRs loci associated with Alzheimer’s disease

Several studies have examined the relationship between VNTRs and LOAD, mostly focussing on specific VNTRs of interest, such as those in the *ABCA7*^81^ and *INS*^82^ genes, and another one near *MUC6,*^83^ or STR in the 5'UTR of the *RELN*^84^ gene (Supplementary Table 1). More recently, genome-wide analyses have extended these findings. A VNTR-based study in 9501 individuals from the Alzheimer’s disease Sequencing Project identified nine novel VNTRs associated with Alzheimer’s disease diagnosis, cognitive decline and brain imaging traits, several of which colocalized with known expression quantitative trait loci (eQTLs) or Alzheimer’s disease risk loci.^85^ In parallel, a large STR association study in approximately 330 000 UK Biobank participants, including clinically diagnosed and proxy Alzheimer’s disease cases, reported 14 independent STR loci associated with Alzheimer’s disease risk.^86^ Most overlapped known SNP-based GWAS regions, but additional STR signals were reported near *SNX32* and *WBS1.*^86^ Interestingly, the *SNX32* gene, which is involved in neuronal differentiation in neuroglial cells in vitro,^87^ was recently highlighted in a proteome-wide association study to be causally involved in Alzheimer’s disease pathogenesis.^88^ In addition, several other loci have been delineated, in which STRs (and not SNPs) represent the lead association signal (ABCA7) or make substantial contributions to SNP-driven GWAS associations (HLA-DRB1, MINDY/ADAM10 and APOE).^86^ The same study also estimated that STRs explain at least 3% of Alzheimer's disease phenotypic variance. Aligning the top STRs with DNA methylation and transcriptome profiles from human brain samples suggests that several STRs may affect gene expression.

### Increased burden of STR expansions in Alzheimer’s disease

Another genome-wide analysis of PCR-free WGS data from 2981 individuals with and without disease showed that Alzheimer’s disease cases carry a greater burden of rare and longer STR expansions across the genome.^66^ Most polymorphic STRs were located in intronic (47.4%) or distal noncoding regions (36.7%), whereas only a small fraction occurred in promoters (14.1%) or coding exons (0.76%).^66^ Statistical analysis revealed that if >30 STR expansions were found, the person was 3.69-fold more likely to have Alzheimer’s disease and also to have more severe neuropathology. Study findings support a polygenic burden model in which the cumulative effect of multiple STR expansions, rather than a single repeat locus, contributes to disease risk, similar to models proposed for autism and schizophrenia.^78-80^

There were 6276 and 5229 unique STR expansions observed in Alzheimer’s disease individuals (AD-STR expansions) and controls, respectively,^66^ with 213 STR expansions present in 5 or more Alzheimer’s disease individuals. AD-STR expansions were slightly but significantly longer, 50.1% of them were dinucleotide repeats, whereas only 2.1% had a repeat unit of 6 bp or longer.^66^ It was demonstrated that although most AD-STR expansions were not protein coding, they were highly enriched near genes implicated in biological processes with known relevance to Alzheimer’s disease pathophysiology, such as ‘neuron projection morphogenesis’ and ‘axon development’.^66^ When AD-STR expansions present within genes (exons, untranslated regions or introns) were examined, enrichment in gene sets related to synaptic function was observed. For example, in the *GRID2* gene they identified 7 STR expansions across 9 individuals with Alzheimer’s disease (only 1 STR in 1 control individual). *GRID2* encodes a subunit of the glutamate receptor and has a recognized role in synaptic transmission.^89^

### Broader genomic instability in Alzheimer’s disease

The widespread distribution of STR expansions across the genome raises the possibility that Alzheimer’s disease is associated with broader genomic instability rather than variation at only a few repeat loci.^66^ Interestingly, prior findings indicate that individuals with Alzheimer’s disease also have a greater burden of rare coding SNVs^90^ and SVs.^13^ Two non-exclusive models have been proposed: either ageing and Alzheimer’s disease-related pathology promote repeat instability, or unstable inherited and/or somatic TR alleles contribute to disease pathogenesis.^66^ Together, these findings suggest that TRs represent an underappreciated layer of Alzheimer’s disease-related genetic variation, acting through both locus-specific and genome-wide effects.

### Challenges of TR detection and genotyping

TR detection remains technically challenging because repetitive regions are difficult to genotype accurately from standard sequencing data. With short-read WGS, many TRs exceed the read length (∼150 bp), leading to partial alignments, stutter noise and imprecise sizing of large alleles.^91^ Reliable analysis therefore depends on sequencing depth, careful quality control (QC) and dedicated tools for genotyping and expansion discovery. Nevertheless, there are tools such as GangSTR^92^ or ExpansionHunterDenovo,^93^ which enable genome-wide STR genotyping in large cohorts, allowing Alzheimer’s disease association studies. Long-read platforms can improve accuracy by sequencing full TRs and flanking regions in single reads, resolving complex structures,^94^ though they are less accessible so far. Importantly, short-read approaches remain effective: a recent study^66^ found strong concordance with PacBio data.

Because TR definitions and reference catalogues differ across tools, no single method captures the full spectrum of repeat variation. Efforts towards consensus callsets and large TR catalogues are ongoing, but many remain biased towards European populations. The TR-Atlas,^95^ currently the most comprehensive reference, profiles ∼860 000 TRs from 338 963 WGS samples (39.5% non-European). Nevertheless, broader inclusion of diverse populations is still needed to reduce ancestry bias and improve assessment of TR variation across populations.

## Somatic mosaicism in Alzheimer’s disease

Somatic mosaicism, defined as genetically distinct cell populations arising from postzygotic mutations, has emerged as a potential contributor to the complexity of Alzheimer’s disease pathogenesis. It is particularly relevant in the brain, where postmitotic neurons accumulate DNA alterations over time.^96^ In addition to sporadic SNVs, mosaic SVs, CNVs and aneuploidies have been reported in post-mortem Alzheimer’s disease brains.^96^ Because these events are often low-frequency, region-specific and cell-type restricted, they are difficult to detect and interpret using standard NGS approaches.^97^

### Biological and clinical relevance of somatic mosaicism in Alzheimer's disease

Brain-specific mosaic SNVs have been identified in Alzheimer’s disease genes, including *PSEN1*, *PSEN2* and *APP*,^98^ and, as well as in additional loci with potential functional relevance in neuronal survival or Alzheimer’s disease-related pathways.^99,100^ Mosaic CNVs, particularly *APP* copy-number gains, may increase gene dosage and promote amyloid pathology.^101^  *APP*-associated mosaic events may also influence phenotype: mosaic *APP* triplication was associated with later disease onset than constitutional triplication,^102^ suggesting that mutation burden and distribution may modify disease severity. Somatic *APP* gene recombination has also been proposed to generate neuronal genomic cDNAs, although this interpretation remains controversial.^103^ More broadly, recurrent chromosomal abnormalities, including mosaic chromosome 21 alternations and X chromosome loss, provide additional evidence for somatic mosaicism in Alzheimer’s disease brains.^104^ However, its precise pathogenic contribution remains unresolved. Together, these findings suggest that somatic mosaic variation may contribute to both biological and clinical heterogeneity in Alzheimer’s disease beyond germline mutations.

### Detection of somatic mosaicism in the NGS era

From an NGS perspective, somatic mosaicism is challenging because true variants must be distinguished from sequencing artefacts, especially at very low variant allele frequencies.^97^ Deep sequencing has identified somatic SNVs in post-mortem Alzheimer’s disease hippocampus,^100^ while high-depth single-cell WGS has shown increased somatic mutational burden in Alzheimer’s disease neurons.^105^ Tools such as Mutect2,^106^ Strelka2,^107^ and SCcaller^108^ support the analysis of ultra-deep and single-cell data, but robust detection still requires stringent filtering, orthogonal validation and careful downstream interpretation.^97,98^

Integration of genomic and single-cell transcriptomic data enables cell-type-aware analysis of somatic variation by linking mosaic variants to specific neuronal and glial populations and by revealing their potential transcriptional consequences.^51,52,105,109^ Such approaches can help distinguish broadly distributed mutations from lineage-restricted or cell-type-specific events, thereby improving interpretation of the functional relevance of somatic variation in Alzheimer’s disease brain tissue.^105,109^ Duplex sequencing further improves the detection of ultra-low-frequency variants by suppressing sequencing artefacts and increasing confidence in rare variant calls.^97,110^ In parallel, emerging long-read approaches may improve resolution of somatic structural variation, repeat-rich regions and complex breakpoints that are difficult to resolve with short-read data alone.^97,110^ Nevertheless, these strategies remain constrained by tissue quality, uneven cellular representation, sequencing depth, cost and the need for specialized analytical workflows and orthogonal validation. Overall, NGS-based and single-cell approaches have substantially expanded the study of somatic mosaicism in Alzheimer’s disease, but major technical and interpretative challenges remain.

## Polygenic risk score (PGS) and polygenic hazard score (PHS) in Alzheimer's disease research

PGS serves to estimate an individual’s lifetime genetic susceptibility to complex polygenic traits or diseases. While PGS estimates an individual’s lifetime genetic predisposition to a disease as a static measure (typically interpreted as a relative risk), PHS incorporates time-to-event (survival) modelling, estimating age-specific risk, thereby providing a dynamic prediction of when the disease is likely to occur rather than just whether it will occur. In Alzheimer’s disease research, PGS and PHS can be used to stratify individuals by genetic risk and support the identification of risk factors, thereby contributing to earlier detection and improved understanding of disease mechanisms.^111^

### PGS and PHS calculation methods and data sources

**Figure 3:**
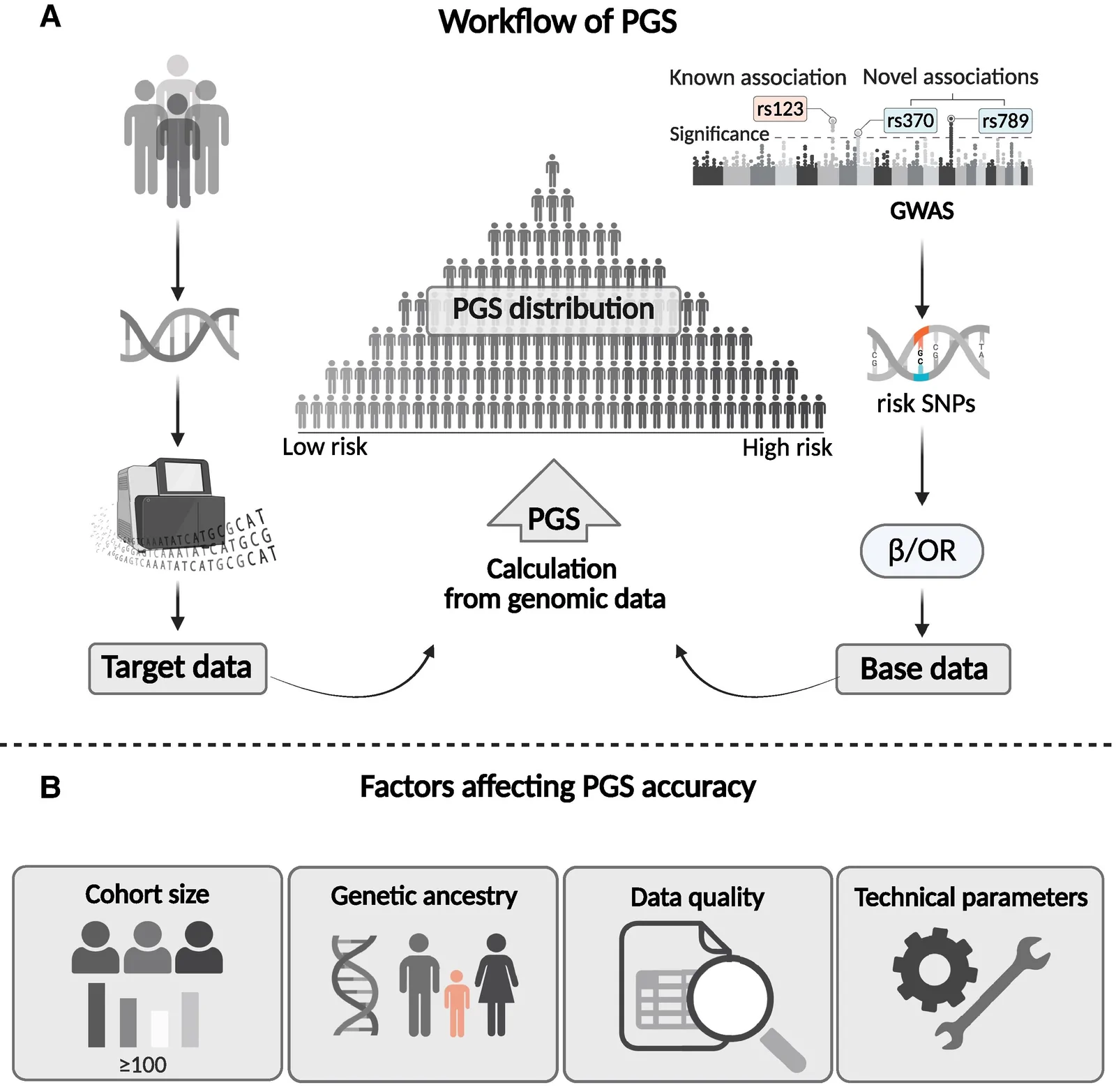
**General workflow for calculating polygenic risk score (PGS) from genomic data and the main factors influencing its accuracy.** (**A**) Workflow of PGS: To perform PGS calculations, base and target data are necessary. The base data include SNPs associated with a complex trait, each carrying an effect size measured by beta (β) for continuous traits or odds ratios (ORs), specifically logOR, for binary traits. These SNPs are typically identified through GWAS and are provided as summary statistics, with a genome-wide significance threshold of *P* < 5 × 10^−8^.^112^ The target data consisted of genotype (list of annotated SNPs) and phenotype (clinical) information from the tested individuals. (**B)** Factors affecting PGS accuracy: A cohort size of ≥100 individuals is recommended.^112^ There is limited transferability of PGS across non-European ancestries, which stems from insufficient diversity in GWASs.^113^ Inaccuracies in effect allele assignment, genome build mismatches, poor ancestry matching, or unresolved ambiguous, duplicate, or mismatching SNPs between base and target data can all contribute to reduced accuracy and introduce systematic bias in the analysis.^112,114^ rs, reference SNP cluster identifier (also called rsID) for SNP variants (created in BioRender. Pös, O. (2026) https://BioRender.com/cv7qzb5).

The schematic PGS workflow is illustrated in Fig. 3A. In general, PGS calculation is based on GWAS-identified SNP datasets that have been shown to be significantly associated with a complex trait, taking into account the effect of these SNPs on the development of a given complex trait in general.^112^ One of the key sources of GWAS data is the NHGRI-EBI GWAS Catalog.^115^ While GWAS focus primarily on common SNPs, currently, emerging studies also explore CNVs, although integration into standard GWAS frameworks remains limited.^116^

The simplest approach to calculate PGS^117^ for an individual is to sum the number of associated alleles (0, 1, or 2) across all associated SNPs, each weighted by its respective effect size. It is possible that multiple SNPs associated with Alzheimer’s disease risk are in linkage disequilibrium (LD), effectively tagging the same underlying risk region. To avoid double-counting this shared genetic signal, thus improving predictive accuracy of the PGS,^118^ commonly used PGS methods apply LD-based clumping and thresholding (C + T), which selects a set of approximately independent variants by excluding those in high LD. Alternatively, newer methods retain all SNPs and use an LD matrix with Bayesian priors (statistical approaches) to shrink and reweight SNP effects in high-LD regions (e.g. LDpred2, PRS-CS, SBayesR), improving genetic risk estimation.^118^ PGS accuracy depends on several factors (Fig. 3B), and QC is crucial for base and target data in order to meet high-quality standards. A valuable source of systematically evaluated and openly accessible PGS scoring files together with standardized metadata required for their reproducible application is the PGS Catalog.^119^ It enables researchers to directly apply previously published PGS models to their own genotype data without the need to reconstruct scores from original GWAS summary statistics, thereby facilitating benchmarking, cross-cohort comparisons and independent validation of genetic risk models.^120^ The PGS Catalog follows FAIR principles (findability, accessibility, interoperability and reusability) and enables automated PGS calculation via the PGS Catalog calculator (pgsc_calc),^120^ while PgsRankRnnotatR tool can be used to prioritize Alzheimer’s disease-related PGS datasets from curated sources (such as PGS Catalog).^121^

Unlike PGS, PHS is established on slightly adjusted standard case-control GWAS summary statistics. PHS incorporates time-to-event (survival) modelling, estimating age-specific risk. For this purpose, Cox Proportional Hazard Regression (Cox regression) analysis is utilized,^122^ to estimate the hazard ratio (HR), a measure between the probability of events in a ‘case’ group compared to the probability of events in a ‘control’ group. Therefore, PHS uses log(HR) as SNP risk allele weights, instead of β used for continuous traits or odds ratios (ORs), specifically ln(OR), for binary traits in PGS (Fig. 3).^123^

### Use of PGS in Alzheimer’s disease risk estimation

The current stage of Alzheimer's disease PGS (AD-PGS) research focuses not only on testing the influence of SNPs in the *APOE* ɛ4 isoform^124,125^ but also on evaluating the influence of SNPs in other associated genes (e.g. *APP*, *GFAP* and *NFL*)^126-128^ and the impact of all common SNPs on changes in PGS distribution.^129,130^ In general, the development of AD-PGS aims to improve the prediction of early risk and the stratification of individuals on the basis of their genetic susceptibility. Therefore, some PGS studies examined the distributions of EOAD and LOAD,^131^ the link between genetic risk and biomarkers,^126,132^ and the influence of non-genetic factors on dementia-related PGS calculations.^133^ However, according to Bellenguez *et al*.,^8^ the developed AD-PGS has shown limited predictive power beyond standard risk factors such as age, sex and *APOE* ɛ4 isoform, as well as poor transferability to non-European populations. Substantially increasing the GWAS sample size might help to overcome these limitations and also reveal otherwise hidden common variants, helping to provide a finite set of SNP variants that are significant for Alzheimer's disease risk determination.^134^ This might also help to clarify whether the condition follows a polygenic or oligogenic genetic architecture.^131^

Moreover, different approaches have to be investigated, since some pathogenic processes associated with the disease may be driven by genetic variants that are not necessarily among those defined directly as the ‘susceptibility to Alzheimer’s disease’ identified by GWAS. For example, GWAS sets based on the levels of different cerebrospinal fluid (CSF) biomarkers (i.e. Aβ and tau protein) have led to identification of independent genetic variants also involved in Alzheimer’s disease susceptibility.^135^ Similarly, a GWAS focused on cognitive performance in over 300 000 individuals identified loci overlapping with Alzheimer’s disease risk regions,^136^ indicating that GWASs capture only part of the genetic variance in Alzheimer’s disease, thus, a PGS derived from GWAS summary statistics alone may not fully capture the total genetic risk in an Alzheimer’s disease patient.

From a methodological point of view, also various PGS calculation approaches have been used for Alzheimer’s disease risk estimation, including C + T (PRSice) and Bayesian approaches (DBSLMM, LDpred2, PRS-CS and SBayesR).^111^

Traditional AD-PGS methods, which estimate disease risk by summing all the identified phenotype-relevant risk alleles, weighted by their effect size, can be effective in ascertaining the overall risk of developing Alzheimer’s disease. Instead, pathway-specific PGS (only variants in genes affecting specific molecular pathways associated with Alzheimer's disease are used) can be better at predicting specific biomarkers of pathology, such as Aβ deposition.^137-139^ A pathway-specific approach can also help to identify the pathways affected to a greater extent in some individuals than in others, eventually enabling a more personalized approach to potential therapy selection in the future.

Another recent study also highlighted interactions between comorbidity-specific PGSs and Alzheimer’s disease, revealing for example shared genetic risks with type 2 diabetes mellitus and epilepsy, both of which are linked to higher prevalence and earlier onset.^140^ These findings suggest that the future development of AD-PGS will likely continue to integrate a broader range of genetic variants beyond the *APOE* ɛ4 isoform, including those associated with biomarkers and cognitive function. The incorporation of biomarker-specific and pathway-based variants, along with non-genetic and comorbidity-related factors, may enhance the predictive power and personalization of Alzheimer’s disease risk assessment.

Thus, AD-PGS is primarily a research tool, but ongoing advances—such as generating pathway-specific PGS,^141^ multi-trait PGS,^142^ functional annotations,^143^ combinations of common and rare variants,^144^ and ancestrally more diverse datasets,^145^ may improve its future clinical application.

### Use of PHS in Alzheimer’s disease risk estimation

Regarding age-dependent Alzheimer’s disease risk estimation, Desikan *et al*.^123^ reported that higher PHS quartiles predict earlier Alzheimer’s disease onset and increased incidence and are strongly associated with neurodegeneration markers and pathological features. Similarly, Tan *et al*.^146^ used an AD-PHS based on 31 selected SNPs to examine its link to pathology and behaviour, and they found associations with amyloid buildup, neurodegeneration and cognitive decline in individuals without an Alzheimer’s disease diagnosis. Similarly, Farhadieh *et al*.^147^ examined whether AD-PHS is linked to biomarkers and cognitive measures and whether it predicts the relationships among *GAP-43*, *NFL* and cognitive decline. They reported that AD-PHS accurately distinguished patients with Alzheimer’s disease, patients with mild cognitive impairment (MCI) and controls.

By building on the foundation of PGS, PHS could provide a more clinically relevant measure of genetic susceptibility. Kauppi *et al*.^148^ demonstrated that PHS significantly predicted progression from MCI to Alzheimer’s disease over a 120-month period and outperformed *APOE* ɛ4 isoforms alone in predictive accuracy. Moreover, integrating PHS with baseline MRI-based brain atrophy scores and/or Mini-Mental State Examination (MMSE) scores further enhanced the ability to predict cognitive decline compared with models lacking PHS. Similarly, Banks *et al*.^149^ tested the predictive strength of applying PHS in combination with composite outcome measures to suggest the design of Alzheimer’s disease clinical trials.

## Alzheimer’s disease protective genetic variants and modifiable factors

There are genetic factors that can be considered protective against Alzheimer’s disease. Interestingly, even genes that are better known for their variants with Alzheimer’s disease-promoting effects can also have other specific variants that, on the contrary, decrease disease development risk.

For example, a rare genetic variant, rs63750847 (p.Ala673Thr), in the *APP* gene affects APP processing by β-secretase. This variant impacts the formation of the β-secretase cleavage site, resulting in a 50% reduction in the production of pathogenic Aβ form and a delay in Aβ aggregation.^150^

Compared to individuals with other *APOE* genotypes, individuals with two copies of the *APOE* ɛ2 isoform were found to have a much lower likelihood of developing Alzheimer's disease. The study by Reiman *et al*.,^151^ which used brain examination to confirm diagnoses, revealed that the odds of having Alzheimer’s disease dementia were reduced by 66% in those with *APOE* ɛ2/ɛ2 compared with those with *APOE* ɛ2/ɛ3, by 87% compared with those with *APOE* ɛ3/ɛ3, and 99.6% compared with those with *APOE* ɛ4/ɛ4 . For individuals with the *APOE* ɛ2/ɛ2 genotype who do develop Alzheimer’s disease, the onset of the disease typically occurs much later in life.^151^

According to a case study by Velasquez *et al*.,^152^ the *APOE* ɛ*3-*Christchurch variant (p.Arg136Ser variant on the background of the *APOE* ɛ*3* isoform) might play a critical role in protecting an individual from EOAD. This protection was observed even though the individual had a genetic predisposition for the disease and a significant buildup of amyloid plaques in their brain.^152^ In addition, two other rare missense variants, *APOE* ɛ*3*-Jacksonville (p.Val236Glu variant on the background of the *APOE* ɛ*3* isoform) and *APOE* ɛ4 isoform with Arg251Gly variant, significantly decrease the risk of Alzheimer’s disease. This protective effect is due to their ability to decrease both tau pathology and amyloid aggregation.^153^

The Klotho-VS allele is a haplotype of the *Klotho* gene containing two missense variants (p.Phe352Va and p.Cys370Ser) in LD. This allele in heterozygous but not homozygous state has been linked to protective phenotypes such as slower cognitive decline, greater cortical volume and reduced amyloid burden. Even *APOE* ɛ4 carriers with a single copy of Klotho-VS have a 1.3 times lower risk of developing Alzheimer’s disease and show a reduced amyloid burden than those who do not carry Klotho-VS.^154^

A case-control study conducted in a Hungarian population identified two variants in the *ABCA1* gene, rs2230805-A and rs2230806-A, that appear to have a protective effect against Alzheimer’s disease. These two variants are in strong LD.^155^ Other genes involved in lipid metabolism, such as *ABCA7* with the variant rs72973581-A^156^ and *SORL1* rs11218343-C,^157^ have the potential to be protective against the development of Alzheimer’s disease. These genes are important for clearing Aβ and maintaining healthy cell membranes. Additionally, the rs142787485-G variant in the *RAB10* gene, which plays a role in the regulation of cellular trafficking, is considered to reduce Alzheimer’s disease risk by up to 1.7-fold.^158^ Some studies also demonstrate that the rs3851179-A variant in the *PICALM* locus is protective against Alzheimer's disease; however, it appears to be restricted to individuals who do not carry the *APOE* ɛ4 isoform.^159,160^ The *TREML2* gene which is expressed by microglia, also contains a protective missense coding variant, rs3747742-C, which results in a serine-to-glycine substitution p.Ser144Gly.^161^ The *PLCG2* gene is also associated with Alzheimer’s disease protection, primarily because a specific variant, p.Pro522Arg, is expressed in microglia and enhances their ability to clear Aβ plaques and modulate inflammatory responses. This protective effect is thought to be mediated by the variant's mild gain-of-function activity, which boosts microglial functions such as phagocytosis and lipid metabolism.^162^ The rare RELN-COLBOS variant (p.His3447Arg) is a gain-of-function change that increases activation of the *RELN* gene downstream of the Dab1 protein. This enhanced signalling reduces tau phosphorylation and confers resilience to the Alzheimer’s disease.^163^

Studying rare protective variants may help to better understand the mechanisms of Alzheimer’s disease resilience and reveal specific biological pathways as potential treatment targets. Similar to PGS for genetic risk, researchers can also use polygenic resilience scores^164^ to combine multiple protective factors, thus potentially improve predictions and guide the development of new combination therapies. These findings also underscore the importance of studying diverse populations, as protective variants can vary across different ethnic groups.

In addition to genetic factors, some modifiable environmental and lifestyle factors can decrease the risk of Alzheimer’s disease development: vascular disease prevention, a healthy lifestyle including sufficient physical activity, good sleep, adequate mental stimuli and a healthy diet.

## Future directions and challenges in Alzheimer’s disease genetics research

### Population specificity of the Alzheimer’s disease genetic risk evaluation and Alzheimer’s disease research data resources

As already mentioned in the text, a key challenge in evaluation of the effect of individual variants on Alzheimer’s disease risk lies in population specificity: allele frequencies, effect sizes, and LD patterns can differ across ancestries. As an example, the impact of *APOE ɛ4*, a well-established risk factor, varies across populations due to differences in surrounding genetic variants (haplotypes), gene–gene interactions and ancestry-specific modifiers.^165^ This means that both monogenic variant associations and PGS prediction are most accurate in populations that are well represented in reference datasets, predominantly individuals of European descent. Understanding population variability of individual Alzheimer's disease risk contributors also in underrepresented populations is essential for improving risk prediction and reducing potential misclassification.

Important roles in overcoming these limitations play a wide range of specialized databases that provide access to genetic, genomic, clinical, imaging and biomarker data related to this disease in different populations. Since 2012, the NIAGADS (The National Institute on Aging Genetics of Alzheimer's Disease Data Storage^166^) platform has provided access to genomic and multi-omics data related to Alzheimer’s disease, Alzheimer’s disease-related dementias and healthy ageing. NIAGADS offers open-access tools, knowledge bases and datasets and serves as the data coordinating centre for the Alzheimer’s disease Sequencing Project (ADSP), managing large-scale sequencing data to support variant discovery and the development of targeted therapies. As of March 2026, NIAGADS collects 145 distinct datasets of various genomic data types (including ≈58 500 whole genome and ≈20 500 exomes), including CRAMs, gVCFs, quality-controlled project level VCFs, and harmonized phenotypes, available for qualified researchers to access. In partnership with NIAGADS, the Alzheimer’s disease Variant Portal (ADVP; https://advp.niagads.org) was developed that provides important insights into population-specific Alzheimer’s disease genetic architecture thanks to easy browsing, searching for and understanding Alzheimer’s disease genetics reported across >80 cohorts and 8 populations.^167^ It harmonizes and annotates genetic associations from more than 200 GWAS publications, making population-specific Alzheimer’s disease findings easily accessible and searchable (7115 Association Records on 956 Unique Loci from 126 Studies). Indeed, one of the aims of the ADVP is to answer the question: what are the population-specific variants associated with Alzheimer’s disease.

Over the past three decades great advances in gene discovery have also been made also by the European Alzheimer’s disease DNA Biobank (EADB^168^), the Alzheimer’s disease European Sequencing Consortium (ADES) and the International Genomics of Alzheimer’s Project (IGAP). The EADB has coordinated sequencing and genetic analysis across numerous European cohorts, and the ADES Consortium is a collaborative initiative focused on Alzheimer’s disease research through genomic sequencing in European populations. While the exact name may vary slightly across publications or projects, it typically involves large-scale WGS or WES efforts to identify genetic risk factors, including rare variants and structural variations, contributing to Alzheimer’s disease. These pioneering studies are summarized elsewhere.^169^ European contributors are also involved in the previously mentioned ADSP Project, although it is primarily a U.S.-based initiative. Both initiatives support data integration and quality control and facilitate collaboration to drive progress in the discovery of genetic markers that can guide the development of targeted treatments and prevention strategies.

### Discovery of true causal gene(s) behind associated variants and other *omics* studies

After the identification of loci associated with Alzheimer’s disease risk, there is a need to discover the true causal gene(s) at these loci, as well as their functional mechanisms. Many associated variants are located far outside the coding region, and it is not easy to understand where they intervene in the disease pathology cascade. However, systematic validation, which is rarely performed due to limitations of time and cost, is needed. Many efforts have been dedicated to the dynamic gene expression process, namely, the epigenome, the transcriptome and the proteome (with a focus on different brain areas), with some important discoveries on various forms of epigenetic regulation, such as DNA methylation, histone post-translational modifications and chromatin accessibility, as well as the transcriptome at single-cell resolution and spatial transcriptomics (recently reviewed^170^).

For example, a transcriptome-wide association study (TWAS) with colocalization analysis, fine-mapping, and additional annotation of AD-GWAS variants was reported to identify 123 genes at known and suggestive Alzheimer’s disease risk loci.^171^ Comparison with human Alzheimer’s disease brain transcriptome data confirmed that many of these candidate genes are dysregulated in Alzheimer’s disease and are correlated with neuropathology. This systematic, integrative approach effectively prioritized genes at GWAS loci and revealed promising Alzheimer’s disease-relevant candidates for further investigation as risk factors or targets for therapeutic intervention.

Recently, a study that used methylation quantitative trait loci (mQTL) showed that brain region-specific DNA methylation is a mediator of genetic risk for Alzheimer’s disease.^172^ A previous study identified multiple CpGs significantly associated with Alzheimer's disease susceptibility, including those linked to the *MAPT* and *LRRFIP2* loci, as well as novel associations with *RITA1.*^172^

Another recent review^173^ highlights the importance of Alzheimer’s disease proteomics to provide insights into disease progression and potential biomarkers. Combining proteomics with genomics enables protein quantitative trait locus (pQTL) analysis in Alzheimer’s disease, linking genetic risk factors directly to protein expression changes. Since proteins are the primary effectors of cellular processes, pQTLs represent a more direct link to disease phenotypes than do eQTLs, which primarily influence mRNA levels. Indeed, discrepancies in RNA-protein abundance (transcriptome-proteome) were observed, suggesting significant post-transcriptional and translational regulation in Alzheimer's disease.^174^ Comparison of the proteome and transcriptome can help to understand RNA-dependent and RNA-independent protein regulation.

Proteomic studies indicate that synaptic disruption is a key driver of Alzheimer’s disease pathology.^174^ Sub-proteomic studies of specific cellular compartments with a focus on the synaptic proteome, have revealed the progressive development of synaptic dysfunction, which begins with early mitochondrial and presynaptic alterations, followed by mid-stage inhibitory synapse impairments and then the selective vulnerability of excitatory synapses in later stages.^175^ Sub-proteomic approaches continue to evolve, now focusing on other critical cellular compartments, such as lysosomes and mitochondria.^173^

### Mosaic changes in neurons and neuronal resilience

Moreover, the widespread presence of mosaic changes in neurons involving different variant types could impact neuronal function and resilience, thus likely playing a critical role in Alzheimer’s disease pathogenesis and extending research interest beyond inherited germline variants. There is a need for sensitive single-cell and tissue-resolved genomic techniques to fully elucidate their mechanistic and clinical significance. A promising direction could be combining single-cell genomics and spatial proteomics.

### Increased mutational potential in Alzheimer's disease

The genomic damage observed in Alzheimer’s disease may also be a consequence of increased mutational potential (i.e. oxidative burden), deficient DNA repair processes (base excision repair), or a combination of both.^176^ Additionally, in relation to the greater STR expansion burden in Alzheimer’s disease, general genomic instability has been proposed as a pathologic characteristic of the genome in Alzheimer’s disease;^66^ thus, it might be interesting to focus more deeply on genes involved in DNA repair and surveillance, which are essential cellular systems that preserve genomic integrity by detecting and correcting DNA damage.

### Individual Alzheimer’s disease risk estimation

As our understanding of Alzheimer’s disease continues to grow, there is an increasing need for tools that can integrate this expanding body of knowledge to estimate individual risk profiles for clinical applications, especially for early detection, targeted interventions, trial enrolment and personal life planning. PGS and PHS are promising in this regard and have made substantial advances in Alzheimer’s disease research; however, a major challenge remains: incorporating diverse types of genetic variation, such as TRs, CNVs and TEs, into risk prediction models. The integration of biomarkers and imaging data into hierarchical pipelines may yield the most clinically actionable models.

These approaches could be improved by using suitable PGS software alongside curated public databases and pipelines, such as the PGS Catalog and pgsc_calc, which facilitate data handling and sharing. Also here, AI-driven biological intelligence could also improve Alzheimer’s disease risk prediction by prioritizing candidate regulatory AD-GWAS loci on the basis of their function. This could lead to the design of more accurate AD-PGS/PHS with stronger mechanistic ties to disease processes.

Current limitations include the modest incremental predictive power of *APOE* ɛ4 isoform, even when considering non-genetic factors, reduced applicability across ancestries and other practical and ethical barriers to widespread use. Taken together, the future application of AD-PGS/PHS requires longitudinal validation, diverse population studies, comprehensive calibration and ethical governance.

### Alzheimer’s disease treatment development

The clinical benefits of individual therapeutic approaches in Alzheimer’s disease remain limited, underlining the complexity of Alzheimer’s disease pathogenesis, since each of them targets a potential disease pathway, and they are not mutually exclusive; they are likely to interact in complex ways. Genomics and related omics technologies can play crucial roles in disentangling these interactions and identifying the genetic underpinnings of each pathway.^8,177^ It is also possible that in the future specific genetic profiles of individual patients might serve to guide the selection of suitable therapy.

### Complexity of the Alzheimer’s disease research

Research in Alzheimer’s disease is inherently complex, encompassing a wide range of study designs and diverse types of data. Emerging ML applications^178^ and AI tools, such as the recently released AlphaGenome,^179^ HyenaDNA^180^ or even the artificial intelligence (AI)-driven chat framework GENEVIC,^181^ could help to better handle the big data phenomenon and identify new patterns of connections within *omics* approaches. A recent review^182^ provides comprehensive insight into the development, application and challenges of large language models (LLMs) used in bioinformatic research.

## Conclusions

As described in this review, Alzheimer’s disease is influenced by a spectrum of different types of small but also larger germline or somatic genetic variants. Rare but highly penetrant variants in ‘deterministic’ genes such as *APP*, *PSEN1* and *PSEN2* are responsible for a proportion of EOAD cases by directly causing the disease through disrupted amyloid processing. Instead, the majority of LOADs (as well as EOADs) are influenced by many common or rare SNPs in genes involved in pathways such as amyloid processing, lipid metabolism, inflammation and synaptic function. In addition to *APOE* ɛ4 isoform, the most significant genetic risk factor so far, other associated SNPs individually confer modest effects; however, together, they contribute to a polygenic risk profile that influences Alzheimer’s disease susceptibility, age of onset and disease progression. Thus, Alzheimer’s disease genetics has moved beyond a monogenic framework to a polygenic framework, which must also be taken into account in the development of risk assessment models. Importantly, emerging evidence from studies focused on CNVs, TRs and TEs suggests that structural and repeat-based variation also play roles in modulating risk, progression, or resilience in Alzheimer’s disease. These variant types represent a previously underexplored source of genetic risk for Alzheimer’s disease, and this must also be taken into account in the development of risk assessment models and could help explain part of the ‘missing heritability’. Thus, future WGS studies are needed, ideally including directly genotyped CNVs and TRs in large datasets from different ancestries, to further delineate their role in the genetic makeup of Alzheimer’s disease.

In addition to the challenge of identifying true causal gene(s) and interpreting the diverse types of associated variants, a central challenge lies in integrating the growing body of knowledge from various areas of Alzheimer’s disease research, including findings from numerous *omics* studies. Emerging AI approaches could help researchers better handle big data phenomena and find new patterns of connections via omics approaches. Such tools can also be useful in possible future clinical application of obtained knowledge in the way of individual Alzheimer's disease risk predictions via PGS/PHS calculations. Nonetheless, the effects of all genetic variants can be modified by other heritable and non-heritable factors. Therefore, together with the development of effective treatment strategies, our efforts should also focus also on increasing awareness among patients and in the general population of modifiable risk factors, such as vascular disease prevention, regular physical activity, adequate sleep, mental stimulation and a healthy diet, to promote habits that lower Alzheimer’s disease risk.

## Supplementary Material

fcag332_Supplementary_Data

## Data Availability

Data sharing is not applicable to this article as no new data were created or analysed in this study.

## References

[1] Ballard C, Gauthier S, Corbett A, Brayne C, Aarsland D, Jones E. Alzheimer’s disease. Lancet. 2011;377(9770):1019–1031. 21371747 10.1016/S0140-6736(10)61349-9

[2] Kamatham PT, Shukla R, Khatri DK, Vora LK. Pathogenesis, diagnostics, and therapeutics for Alzheimer’s disease: Breaking the memory barrier. Ageing Res Rev. 2024;101:102481. 39236855 10.1016/j.arr.2024.102481

[3] Knopman DS, Amieva H, Petersen RC, et al Alzheimer disease. Nat Rev Dis Primers. 2021;7(1):33. 33986301 10.1038/s41572-021-00269-y PMC8574196

[4] Selkoe DJ, Hardy J. The amyloid hypothesis of Alzheimer’s disease at 25 years. EMBO Mol Med. 2016;8:595–608. 27025652 10.15252/emmm.201606210 PMC4888851

[5] Almansoub HAMM, Tang H, Wu Y, et al Tau abnormalities and the potential therapy in Alzheimer’s disease. J Alzheimers Dis. 2019;67(1):13–33. 30507581 10.3233/JAD-180868

[6] Schreiner TG, Schreiner OD, Adam M, Popescu BO. The roles of the amyloid beta monomers in physiological and pathological conditions. Biomedicines. 2023;11(5):1411. 37239082 10.3390/biomedicines11051411 PMC10216198

[7] Gatz M, Reynolds CA, Fratiglioni L, et al Role of genes and environments for explaining Alzheimer disease. Arch Gen Psychiatry. 2006;63(2):168–174. 16461860 10.1001/archpsyc.63.2.168

[8] Bellenguez C, Küçükali F, Jansen IE, et al New insights into the genetic etiology of Alzheimer’s disease and related dementias. Nat Genet. 2022;54(4):412–436. 35379992 10.1038/s41588-022-01024-z PMC9005347

[9] Lambert JC, Ramirez A, Grenier-Boley B, Bellenguez C. Step by step: Towards a better understanding of the genetic architecture of Alzheimer’s disease. Mol Psychiatry. 2023;28(7):2716–2727. 37131074 10.1038/s41380-023-02076-1 PMC10615767

[10] Wang LS, Naj AC, Graham RR, et al Rarity of the Alzheimer disease-protective APP A673T variant in the United States. JAMA Neurol. 2015;72(2):209–216. 25531812 10.1001/jamaneurol.2014.2157 PMC4324097

[11] Wingo TS, Lah JJ, Levey AI, Cutler DJ. Autosomal recessive causes likely in early-onset Alzheimer disease. Arch Neurol. 2012;69(1):59–64. 21911656 10.1001/archneurol.2011.221 PMC3332307

[12] Zhou X, Chen Y, Ip FCF, et al Deep learning-based polygenic risk analysis for Alzheimer’s disease prediction. Commun Med (Lond). 2023;3(1):49. 37024668 10.1038/s43856-023-00269-x PMC10079691

[13] Wang H, Dombroski BA, Cheng PL, et al Structural variation detection and association analysis of whole-genome-sequence data from 16,543 Alzheimer’s disease sequencing project subjects. Alzheimers Dement. 2025;21(6):e70277. 40528303 10.1002/alz.70277 PMC12173831

[14] Andrews SJ, Renton AE, Fulton-Howard B, Podlesny-Drabiniok A, Marcora E, Goate AM. The complex genetic architecture of Alzheimer’s disease: Novel insights and future directions. EBioMedicine. 2023;90:104511. 36907103 10.1016/j.ebiom.2023.104511 PMC10024184

[15] Youssoufian H, Pyeritz RE. Mechanisms and consequences of somatic mosaicism in humans. Nat Rev Genet. 2002;3(10):748–758. 12360233 10.1038/nrg906

[16] De Coster W, Weissensteiner MH, Sedlazeck FJ. Towards population-scale long-read sequencing. Nat Rev Genet. 2021;22(9):572–587. 34050336 10.1038/s41576-021-00367-3 PMC8161719

[17] Espinosa E, Bautista R, Larrosa R, Plata O. Advancements in long-read genome sequencing technologies and algorithms. Genomics. 2024;116(3):110842. 38608738 10.1016/j.ygeno.2024.110842

[18] Ibañez K, Polke J, Hagelstrom RT, et al Whole genome sequencing for the diagnosis of neurological repeat expansion disorders in the UK: A retrospective diagnostic accuracy and prospective clinical validation study. Lancet Neurol. 2022;21(3):234–245. 35182509 10.1016/S1474-4422(21)00462-2 PMC8850201

[19] Leung YY, Lee WP, Kuzma AB, et al Alzheimer’s disease sequencing project release 4 whole genome sequencing dataset. Alzheimers Dement. 2025;21(5):e70237. 40407102 10.1002/alz.70237 PMC12100500

[20] Karagas N, Young JE, Blue EE, Jayadev S. The spectrum of genetic risk in Alzheimer disease. Neurol Genet. 2025;11(1):e200224. 39885961 10.1212/NXG.0000000000200224 PMC11781270

[21] Coppola G, Chinnathambi S, Lee JJ, et al Evidence for a role of the rare p.A152T variant in MAPT in increasing the risk for FTD-spectrum and Alzheimer’s diseases. Hum Mol Genet. 2012;21(15):3500–3512. 22556362 10.1093/hmg/dds161 PMC3392107

[22] Lumsden AL, Mulugeta A, Zhou A, Hyppönen E. Apolipoprotein E (APOE) genotype-associated disease risks: A phenome-wide, registry-based, case-control study utilising the UK biobank. EBioMedicine. 2020;59:102954. 32818802 10.1016/j.ebiom.2020.102954 PMC7452404

[23] Blanchard JW, Akay LA, Davila-Velderrain J, et al APOE4 impairs myelination via cholesterol dysregulation in oligodendrocytes. Nature. 2022;611(7937):769–779. 36385529 10.1038/s41586-022-05439-w PMC9870060

[24] Farrer LA, Cupples LA, Haines JL, et al Effects of age, sex, and ethnicity on the association between apolipoprotein E genotype and Alzheimer disease: A meta-analysis. APOE and Alzheimer Disease Meta Analysis Consortium. JAMA. 1997;278:1349–1356. 9343467

[25] Uffelmann E, Wightman DP, Bahrami S, et al Genomic analyses reveal new insights into Alzheimer’s disease. medRxiv. [Preprint] doi:10.1101/2025.10.10.25337470

[26] Krishnamurthy HK, Jayaraman V, Krishna K, et al An overview of the genes and biomarkers in Alzheimer’s disease. Ageing Res Rev. 2025;104:102599. 39612989 10.1016/j.arr.2024.102599

[27] Andersen P, de Waal M, Monti G, et al Domain mapping of disease mutations reveals pathogenic SORL1 variants in Alzheimer’s disease. Mol Neurodegeneration. 2025;20:122. 10.1186/s13024-025-00907-z PMC12667055 41327266

[28] Pottier C, Hannequin D, Coutant S, et al High frequency of potentially pathogenic SORL1 mutations in autosomal dominant early-onset Alzheimer disease. Mol Psychiatry. 2012;17(9):875–879. 22472873 10.1038/mp.2012.15

[29] Guerreiro R, Wojtas A, Bras J, et al TREM2 variants in Alzheimer’s disease. N Engl J Med. 2013;368(2):117–127. 23150934 10.1056/NEJMoa1211851 PMC3631573

[30] Steinberg S, Stefansson H, Jonsson T, et al Loss-of-function variants in ABCA7 confer risk of Alzheimer’s disease. Nat Genet. 2015;47(5):445–447. 25807283 10.1038/ng.3246

[31] De Deyn L, Sleegers K. The impact of rare genetic variants on Alzheimer disease. Nat Rev Neurol. 2025;21(3):127–139. 39905212 10.1038/s41582-025-01062-1

[32] Richards S, Aziz N, Bale S, et al Standards and guidelines for the interpretation of sequence variants: A joint consensus recommendation of the American College of Medical Genetics and Genomics and the Association for Molecular Pathology. Genet Med. 2015;17(5):405–424. 25741868 10.1038/gim.2015.30 PMC4544753

[33] Karczewski KJ, Francioli LC, Tiao G, et al The mutational constraint spectrum quantified from variation in 141,456 humans. Nature. 2020;581(7809):434–443. 32461654 10.1038/s41586-020-2308-7 PMC7334197

[34] Guez J, Goodrich JK, Moldovan MA, et al Integrating 730,947 exome sequences with clinical literature improves gene discovery. medRxiv. [Preprint] doi:10.64898/2026.03.23.26349081

[35] Landrum MJ, Lee JM, Benson M, et al ClinVar: Improving access to variant interpretations and supporting evidence. Nucleic Acids Res. 2018;46(D1):D1062–D1067. 29165669 10.1093/nar/gkx1153 PMC5753237

[36] Amberger JS, Bocchini CA, Schiettecatte F, Scott AF, Hamosh A. OMIM.org: Online Mendelian inheritance in man (OMIM®), an online catalog of human genes and genetic disorders. Nucleic Acids Res. 2015;43(Database issue):D789–D798. 25428349 10.1093/nar/gku1205 PMC4383985

[37] Huang J, McLean GR, Franke A. Twenty years of genome-wide association studies: Health translation challenges and AI opportunities. Eur J Hum Genet. 2025;33(12):1579–1584. 41087731 10.1038/s41431-025-01951-5 PMC12669238

[38] Uffelmann E, Huang QQ, Munung NS, et al Genome-wide association studies. Nat Rev Methods Primers. 2021;1(1):1–21.

[39] Quick C, Anugu P, Musani S, et al Sequencing and imputation in GWAS: Cost-effective strategies to increase power and genomic coverage across diverse populations. Genet Epidemiol. 2020;44(6):537–549. 32519380 10.1002/gepi.22326 PMC7449570

[40] Li B, Leal SM. Methods for detecting associations with rare variants for common diseases: Application to analysis of sequence data. Am J Hum Genet. 2008;83(3):311–321. 18691683 10.1016/j.ajhg.2008.06.024 PMC2842185

[41] Wu MC, Lee S, Cai T, Li Y, Boehnke M, Lin X. Rare-variant association testing for sequencing data with the sequence kernel association test. Am J Hum Genet. 2011;89(1):82–93. 21737059 10.1016/j.ajhg.2011.05.029 PMC3135811

[42] Lee S, Emond MJ, Bamshad MJ, et al Optimal unified approach for rare-variant association testing with application to small-sample case-control whole-exome sequencing studies. Am J Hum Genet. 2012;91(2):224–237. 22863193 10.1016/j.ajhg.2012.06.007 PMC3415556

[43] Pös O, Radvanszky J, Buglyó G, et al DNA copy number variation: Main characteristics, evolutionary significance, and pathological aspects. Biomed J. 2021;44(5):548–559. 34649833 10.1016/j.bj.2021.02.003 PMC8640565

[44] Rovelet-Lecrux A, Hannequin D, Raux G, et al APP locus duplication causes autosomal dominant early-onset Alzheimer disease with cerebral amyloid angiopathy. Nat Genet. 2006;38(1):24–26. 16369530 10.1038/ng1718

[45] Crook R, Verkkoniemi A, Perez-Tur J, et al A variant of Alzheimer’s disease with spastic paraparesis and unusual plaques due to deletion of exon 9 of presenilin 1. Nat Med. 1998;4(4):452–455. 9546792 10.1038/nm0498-452

[46] Brouwers N, Van Cauwenberghe C, Engelborghs S, et al Alzheimer risk associated with a copy number variation in the complement receptor 1 increasing C3b/C4b binding sites. Mol Psychiatry. 2012;17(2):223–233. 21403675 10.1038/mp.2011.24 PMC3265835

[47] Lambert JC, Heath S, Even G, et al Genome-wide association study identifies variants at CLU and CR1 associated with Alzheimer’s disease. Nat Genet. 2009;41(10):1094–1099. 19734903 10.1038/ng.439

[48] Swaminathan S, Kim S, Shen L, et al Genomic copy number analysis in Alzheimer’s disease and mild cognitive impairment: An ADNI study. Int J Alzheimers Dis. 2011;2011:729478. 21660214 10.4061/2011/729478 PMC3109875

[49] Quenez O, Schramm C, Cassinari K, et al Exome analysis of 22,319 individuals links extremely rare copy-number variants and 22q11.21 dosage to Alzheimer risk. Am J Hum Genet. Published online August 26, 2026. 10.1016/j.ajhg.2026.07.013 42648288

[50] Butcher NJ, Horne MK, Mellick GD, et al Sulfotransferase 1A3/4 copy number variation is associated with neurodegenerative disease. Pharmacogenomics J. 2018;18(2):209–214. 28374858 10.1038/tpj.2017.4

[51] Ming C, Wang M, Wang Q, et al Whole genome sequencing-based copy number variations reveal novel pathways and targets in Alzheimer’s disease. Alzheimers Dement. 2022;18(10):1846–1867. 34918867 10.1002/alz.12507 PMC9264340

[52] Lee WP, Tucci AA, Conery M, et al Copy number variation identification on 3,800 Alzheimer’s disease whole genome sequencing data from the Alzheimer's disease sequencing project. Front Genet. 2021;12:752390. 34804120 10.3389/fgene.2021.752390 PMC8599981

[53] Byman E, Nägga K, Gustavsson AM, et al Alpha-amylase 1A copy number variants and the association with memory performance and Alzheimer’s dementia. Alzheimers Res Ther. 2020;12(1):158. 33220711 10.1186/s13195-020-00726-y PMC7680592

[54] Hao Y, Li C, Wang H, Ming C. Effects of copy number variations on longevity in late-onset Alzheimer’s disease patients: Insights from a causality network analysis. Front Aging Neurosci. 2023;15:1241412. 38020759 10.3389/fnagi.2023.1241412 PMC10652415

[55] Li Y, Shaw CA, Sheffer I, et al Integrated copy number and gene expression analysis detects a CREB1 association with Alzheimer’s disease. Transl Psychiatry. 2012;2(11):e192. 23168992 10.1038/tp.2012.119 PMC3565761

[56] Swaminathan S, Shen L, Kim S, et al Analysis of copy number variation in Alzheimer’s disease: The NIALOAD/NCRAD family study. Curr Alzheimer Res. 2012;9(7):801–814. 22486522 10.2174/156720512802455331 PMC3500615

[57] Wang H, Wang LS, Schellenberg G, Lee WP. The role of structural variations in Alzheimer’s disease and other neurodegenerative diseases. Front Aging Neurosci. 2022;14:1073905. 36846102 10.3389/fnagi.2022.1073905 PMC9944073

[58] Zheng X, Demirci FY, Barmada MM, et al Genome-wide copy-number variation study of psychosis in Alzheimer’s disease. Transl Psychiatry. 2015;5(6):e574. 26035058 10.1038/tp.2015.64 PMC4490277

[59] Zhang Y, Liu W, Duan J. On the core segmentation algorithms of copy number variation detection tools. Brief Bioinform. 2024;25(2):bbae022. 38340093 10.1093/bib/bbae022 PMC10858679

[60] Vialle RA, de Paiva Lopes K, Li Y, et al Structural variants linked to Alzheimer’s disease and other common age-related clinical and neuropathologic traits. Genome Med. 2025;17(1):20. 40038788 10.1186/s13073-025-01444-6 PMC11881306

[61] Olivucci G, Iovino E, Innella G, Turchetti D, Pippucci T, Magini P. Long read sequencing on its way to the routine diagnostics of genetic diseases. Front Genet. 2024;15:1374860. 38510277 10.3389/fgene.2024.1374860 PMC10951082

[62] Gurbich TA, Ilinsky VV. ClassifyCNV: A tool for clinical annotation of copy-number variants. Sci Rep. 2020;10(1):20375. 33230148 10.1038/s41598-020-76425-3 PMC7683568

[63] Turan ZG, Richter V, Bochmann J, et al Somatic copy number variant load in neurons of healthy controls and Alzheimer’s disease patients. Acta Neuropathol Commun. 2022;10(1):175. 36451207 10.1186/s40478-022-01452-2 PMC9714068

[64] de Koning APJ, Gu W, Castoe TA, Batzer MA, Pollock DD. Repetitive elements may comprise over two-thirds of the human genome. PLoS Genet. 2011;7(12):e1002384. 22144907 10.1371/journal.pgen.1002384 PMC3228813

[65] Chénais B . Transposable elements and human diseases: Mechanisms and implication in the response to environmental pollutants. Int J Mol Sci. 2022;23(5):2551. 35269693 10.3390/ijms23052551 PMC8910135

[66] Guo MH, Lee WP, Vardarajan B, Schellenberg GD, Phillips-Cremins JE. Polygenic burden of short tandem repeat expansions promotes risk for Alzheimer’s disease. Nat Commun. 2025;16(1):1126. 39875385 10.1038/s41467-025-56400-0 PMC11775329

[67] van Bree EJ, Guimarães RLFP, Lundberg M, et al A hidden layer of structural variation in transposable elements reveals potential genetic modifiers in human disease-risk loci. Genome Res. 2022;32(4):656–670. 35332097 10.1101/gr.275515.121 PMC8997352

[68] Larsen PA, Lutz MW, Hunnicutt KE, et al The Alu neurodegeneration hypothesis: A primate-specific mechanism for neuronal transcription noise, mitochondrial dysfunction, and manifestation of neurodegenerative disease. Alzheimers Dement. 2017;13(7):828–838. 28242298 10.1016/j.jalz.2017.01.017 PMC6647845

[69] Feng Y, Yang X, Hou Y, et al Widespread transposable element dysregulation in human aging brains with Alzheimer’s disease. Alzheimers Dement. 2024;20(11):7495–7517. 39356058 10.1002/alz.14164 PMC11567813

[70] Guo C, Jeong HH, Hsieh YC, et al Tau activates transposable elements in Alzheimer’s disease. Cell Rep. 2018;23(10):2874–2880. 29874575 10.1016/j.celrep.2018.05.004 PMC6181645

[71] Bilgrav Saether K, Eisfeldt J. Detecting transposable elements in long-read genomes using sTELLeR. Bioinformatics. 2024;40(11):btae686. 39558574 10.1093/bioinformatics/btae686 PMC11601167

[72] Groza C, Chen X, Wheeler TJ, Bourque G, Goubert C. A unified framework to analyze transposable element insertion polymorphisms using graph genomes. Nat Commun. 2024;15(1):8915. 39414821 10.1038/s41467-024-53294-2 PMC11484939

[73] Malik I, Kelley CP, Wang ET, Todd PK. Molecular mechanisms underlying nucleotide repeat expansion disorders. Nat Rev Mol Cell Biol. 2021;22(9):589–607. 34140671 10.1038/s41580-021-00382-6 PMC9612635

[74] Sulovari A, Li R, Audano PA, et al Human-specific tandem repeat expansion and differential gene expression during primate evolution. Proc Natl Acad Sci U S A. 2019;116(46):23243–23253. 31659027 10.1073/pnas.1912175116 PMC6859368

[75] Depienne C, Mandel JL. 30 years of repeat expansion disorders: What have we learned and what are the remaining challenges? Am J Hum Genet. 2021;108(5):764–785. 33811808 10.1016/j.ajhg.2021.03.011 PMC8205997

[76] Gymrek M, Willems T, Guilmatre A, et al Abundant contribution of short tandem repeats to gene expression variation in humans. Nat Genet. 2016;48(1):22–29. 26642241 10.1038/ng.3461 PMC4909355

[77] Jakubosky D, D’Antonio M, Bonder MJ, et al Properties of structural variants and short tandem repeats associated with gene expression and complex traits. Nat Commun. 2020;11(1):2927. 32522982 10.1038/s41467-020-16482-4 PMC7286898

[78] Mitra I, Huang B, Mousavi N, et al Patterns of de novo tandem repeat mutations and their role in autism. Nature. 2021;589(7841):246–250. 33442040 10.1038/s41586-020-03078-7 PMC7810352

[79] Wen J, Trost B, Engchuan W, et al Rare tandem repeat expansions associate with genes involved in synaptic and neuronal signaling functions in schizophrenia. Mol Psychiatry. 2023;28(1):475–482. 36380236 10.1038/s41380-022-01857-4 PMC9812781

[80] Trost B, Engchuan W, Nguyen CM, et al Genome-wide detection of tandem DNA repeats that are expanded in autism. Nature. 2020;586(7827):80–86. 32717741 10.1038/s41586-020-2579-z PMC9348607

[81] Duchateau L, Küçükali F, De Roeck A, et al CSF biomarker analysis of ABCA7 mutation carriers suggests altered APP processing and reduced inflammatory response. Alzheimers Res Ther. 2023;15(1):195. 37946268 10.1186/s13195-023-01338-y PMC10634183

[82] Majores M, Kölsch H, Bagli M, et al The insulin gene VNTR polymorphism in Alzheimer’s disease: Results of a pilot study. J Neural Transm (Vienna). 2002;109(7-8):1029–1034. 12111440 10.1007/s007020200086

[83] Katsumata Y, Fardo DW, Bachstetter AD, et al Alzheimer disease pathology-associated polymorphism in a Complex Variable number of tandem repeat region within the MUC6 gene, near the AP2A2 gene. J Neuropathol Exp Neurol. 2020;79(1):3–21. 31748784 10.1093/jnen/nlz116 PMC8204704

[84] Seripa D, Matera MG, Franceschi M, et al The RELN locus in Alzheimer’s disease. J Alzheimers Dis. 2008;14(3):335–344. 18599960 10.3233/jad-2008-14308

[85] Heath A, McNerney MW, Yesavage J. Whole genome Variable number tandem repeat analysis in Alzheimer disease. Neurol Genet. 2025;11(2):e200241. 39980902 10.1212/NXG.0000000000200241 PMC11839231

[86] Gmelin D, Ohlei O, Aslam MM, et al GWAS on short tandem repeats identifies genetic mechanisms in Alzheimer’s disease. Nat Commun. 2026;17:4968. 42243151 10.1038/s41467-026-73902-7 PMC13237113

[87] Sugatha J, Priya A, Raj P, et al Insights into cargo sorting by SNX32 and its role in neurite outgrowth. Elife. 2023;12:e84396. 37158588 10.7554/eLife.84396 PMC10219652

[88] Wingo AP, Liu Y, Gerasimov ES, et al Integrating human brain proteomes with genome-wide association data implicates new proteins in Alzheimer’s disease pathogenesis. Nat Genet. 2021;53(2):143–146. 33510477 10.1038/s41588-020-00773-z PMC8130821

[89] Kashiwabuchi N, Ikeda K, Araki K, et al Impairment of motor coordination, Purkinje cell synapse formation, and cerebellar long-term depression in GluRδ2 mutant mice. Cell. 1995;81(2):245–252. 7736576 10.1016/0092-8674(95)90334-8

[90] Kunkle BW, Grenier-Boley B, Sims R, et al Genetic meta-analysis of diagnosed Alzheimer’s disease identifies new risk loci and implicates Aβ, tau, immunity and lipid processing. Nat Genet. 2019;51(3):414–430. 30820047 10.1038/s41588-019-0358-2 PMC6463297

[91] Tanudisastro HA, Deveson IW, Dashnow H, MacArthur DG. Sequencing and characterizing short tandem repeats in the human genome. Nat Rev Genet. 2024;25(7):460–475. 38366034 10.1038/s41576-024-00692-3

[92] Mousavi N, Shleizer-Burko S, Yanicky R, Gymrek M. Profiling the genome-wide landscape of tandem repeat expansions. Nucleic Acids Res. 2019;47(15):e90. 31194863 10.1093/nar/gkz501 PMC6735967

[93] Dolzhenko E, Bennett MF, Richmond PA, et al ExpansionHunter denovo: A computational method for locating known and novel repeat expansions in short-read sequencing data. Genome Biol. 2020;21(1):102. 32345345 10.1186/s13059-020-02017-z PMC7187524

[94] Ziaei Jam H, Zook JM, Javadzadeh S, Park J, Sehgal A, Gymrek M. LongTR: Genome-wide profiling of genetic variation at tandem repeats from long reads. Genome Biol. 2024;25(1):176. 38965568 10.1186/s13059-024-03319-2 PMC11229021

[95] Cui Y, Ye W, Li JS, et al A genome-wide spectrum of tandem repeat expansions in 338,963 humans. Cell. 2024;187(9):2336–2341.e5. 38582080 10.1016/j.cell.2024.03.004 PMC11065452

[96] Iourov IY, Vorsanova SG, Kurinnaia OS, Kutsev SI, Yurov YB. Somatic mosaicism in the diseased brain. Mol Cytogenet. 2022;15(1):45. 36266706 10.1186/s13039-022-00624-y PMC9585840

[97] Menon V, Brash DE. Next-generation sequencing methodologies to detect low-frequency mutations: “Catch me if you can”. Mutat Res Rev Mutat Res. 2023;792:108471. 37716438 10.1016/j.mrrev.2023.108471 PMC10843083

[98] Sala Frigerio C, Lau P, Troakes C, et al On the identification of low allele frequency mosaic mutations in the brains of Alzheimer’s disease patients. Alzheimers Dement. 2015;11(11):1265–1276. 25937274 10.1016/j.jalz.2015.02.007

[99] Helgadottir HT, Lundin P, Wallén Arzt E, Lindström AK, Graff C, Eriksson M. Somatic mutation that affects transcription factor binding upstream of CD55 in the temporal cortex of a late-onset Alzheimer disease patient. Hum Mol Genet. 2019;28(16):2675–2685. 31216356 10.1093/hmg/ddz085 PMC6688063

[100] Park JS, Lee J, Jung ES, et al Brain somatic mutations observed in Alzheimer’s disease associated with aging and dysregulation of tau phosphorylation. Nat Commun. 2019;10(1):3090. 31300647 10.1038/s41467-019-11000-7 PMC6626023

[101] Bushman DM, Kaeser GE, Siddoway B, et al Genomic mosaicism with increased amyloid precursor protein (APP) gene copy number in single neurons from sporadic Alzheimer’s disease brains. Elife. 2015:4:e05116. 25650802 10.7554/eLife.05116 PMC4337608

[102] Ehn E, Eisfeldt J, Laffita-Mesa JM, et al A de novo, mosaic and complex chromosome 21 rearrangement causes APP triplication and familial autosomal dominant early onset Alzheimer disease. Sci Rep. 2025;15(1):2912. 39849058 10.1038/s41598-025-86645-0 PMC11759332

[103] Lee MH, Siddoway B, Kaeser GE, et al Somatic APP gene recombination in Alzheimer’s disease and normal neurons. Nature. 2018;563(7733):639–645. 30464338 10.1038/s41586-018-0718-6 PMC6391999

[104] Yurov YB, Vorsanova SG, Liehr T, Kolotii AD, Iourov IY. X chromosome aneuploidy in the Alzheimer’s disease brain. Mol Cytogenet. 2014;7(1):20. 24602248 10.1186/1755-8166-7-20 PMC3995993

[105] Kousi M, Boix C, Park YP, et al Single-cell mosaicism analysis reveals cell-type-specific somatic mutational burden in Alzheimer’s Dementia. bioRxiv. [Preprint] doi:10.1101/2022.04.21.489103

[106] Benjamin D, Sato T, Cibulskis K, Getz G, Stewart C, Lichtenstein L. Calling Somatic SNVs and Indels with Mutect2. bioRxiv. [Preprint] doi:10.1101/861054

[107] Kim S, Scheffler K, Halpern AL, et al Strelka2: Fast and accurate calling of germline and somatic variants. Nat Methods. 2018;15(8):591–594. 30013048 10.1038/s41592-018-0051-x

[108] Dong X, Zhang L, Milholland B, et al Accurate identification of single-nucleotide variants in whole-genome-amplified single cells. Nat Methods. 2017;14(5):491–493. 28319112 10.1038/nmeth.4227 PMC5408311

[109] Zhang M, Bouland GA, Holstege H, Reinders MJT. Identifying aging and Alzheimer disease-associated somatic variations in excitatory neurons from the human frontal Cortex. Neurol Genet. 2023;9(3):e200066. 37123987 10.1212/NXG.0000000000200066 PMC10136684

[110] Abascal F, Harvey LMR, Mitchell E, et al Somatic mutation landscapes at single-molecule resolution. Nature. 2021;593(7859):405–410. 33911282 10.1038/s41586-021-03477-4

[111] Clark K, Leung YY, Lee WP, Voight B, Wang LS. Polygenic risk scores in Alzheimer’s disease genetics: Methodology, applications, inclusion, and diversity. J Alzheimers Dis. 2022;89:1–12. 35848019 10.3233/JAD-220025 PMC9484091

[112] Choi SW, Mak TSH, O’Reilly PF. Tutorial: A guide to performing polygenic risk score analyses. Nat Protoc. 2020;15(9):2759–2772. 32709988 10.1038/s41596-020-0353-1 PMC7612115

[113] Kachuri L, Chatterjee N, Hirbo J, et al Principles and methods for transferring polygenic risk scores across global populations. Nat Rev Genet. 2024;25(1):8–25. 37620596 10.1038/s41576-023-00637-2 PMC10961971

[114] Mostafavi H, Harpak A, Agarwal I, Conley D, Pritchard JK, Przeworski M. Variable prediction accuracy of polygenic scores within an ancestry group. Elife. 2020;9:e48376. 31999256 10.7554/eLife.48376 PMC7067566

[115] Cerezo M, Sollis E, Ji Y, et al The NHGRI-EBI GWAS catalog: Standards for reusability, sustainability and diversity. Nucleic Acids Res. 2025;53(D1):D998–D1005. 39530240 10.1093/nar/gkae1070 PMC11701593

[116] Harris L, McDonagh EM, Zhang X, et al Genome-wide association testing beyond SNPs. Nat Rev Genet. 2025;26(3):156–170. 39375560 10.1038/s41576-024-00778-y PMC12329314

[117] Talmud PJ, Cooper JA, Palmen J, et al Chromosome 9p21.3 coronary heart disease locus genotype and prospective risk of CHD in healthy middle-aged men. Clin Chem. 2008;54(3):467–474. 18250146 10.1373/clinchem.2007.095489

[118] Modeling linkage disequilibrium increases accuracy of polygenic risk scores. Am J Hum Genet. 2015;97(4):576–592. 26430803 10.1016/j.ajhg.2015.09.001 PMC4596916

[119] Catalog PGS . PGS catalog—the polygenic score catalog. Accessed 14 August 2025. https://www.pgscatalog.org/

[120] Lambert SA, Wingfield B, Gibson JT, et al Enhancing the polygenic score catalog with tools for score calculation and ancestry normalization. Nat Genet. 2024;56(10):1989–1994. 39327485 10.1038/s41588-024-01937-x PMC12041910

[121] Mwesigwa S, Dai Y, Enduru N, Zhao Z. Enhancing the utility of polygenic scores in Alzheimer’s disease through systematic curation and annotation. Front Genet. 2025;16:1507395. 39967687 10.3389/fgene.2025.1507395 PMC11832703

[122] Cox DR . Regression models and life-tables. J R Stat Soc Ser B Stat Methodol. 1972;34(2):187–202.

[123] Desikan RS, Fan CC, Wang Y, et al Genetic assessment of age-associated Alzheimer disease risk: Development and validation of a polygenic hazard score. PLoS Med. 2017;14(3):e1002258. 28323831 10.1371/journal.pmed.1002258 PMC5360219

[124] Najar J, van der Lee SJ, Joas E, et al Polygenic risk scores for Alzheimer’s disease are related to dementia risk in APOE ɛ4 negatives. Alzheimers Dement (Amst). 2021;13(1):e12142. 33532541 10.1002/dad2.12142 PMC7821873

[125] Huq AJ, Fulton-Howard B, Riaz M, et al Polygenic score modifies risk for Alzheimer's disease in *APOE* ɛ4 homozygotes at phenotypic extremes. Alzheimers Dement (Amst). 2021;13(1):e12226. 34386572 10.1002/dad2.12226 PMC8339682

[126] Jiao B, Xiao X, Yuan Z, et al Associations of risk genes with onset age and plasma biomarkers of Alzheimer’s disease: A large case-control study in mainland China. Neuropsychopharmacology. 2022;47(5):1121–1127. 35001095 10.1038/s41386-021-01258-1 PMC8938514

[127] Porter T, Burnham SC, Milicic L, et al Utility of an Alzheimer’s disease risk-weighted polygenic risk score for predicting rates of cognitive decline in preclinical Alzheimer's disease: A prospective longitudinal study. J Alzheimers Dis. 2018;66(3):1193–1211. 30412495 10.3233/JAD-180713

[128] Xu Y, Qiao M, Gunasekaran TI, et al Evaluating polygenic risk score prediction performance for Alzheimer’s disease in a population-based Hispanic cohort using single- and multi-ancestry models. Lancet Reg Health Am. 2025;49:101198. 40747112 10.1016/j.lana.2025.101198 PMC12312067

[129] de Rojas I, Moreno-Grau S, Tesi N, et al Common variants in Alzheimer’s disease and risk stratification by polygenic risk scores. Nat Commun. 2021;12(1):3417. 34099642 10.1038/s41467-021-22491-8 PMC8184987

[130] Altmann A, Scelsi MA, Shoai M, et al A comprehensive analysis of methods for assessing polygenic burden on Alzheimer's disease pathology and risk beyond *APOE*. Brain Commun. 2020;2(1):fcz047. 32226939 10.1093/braincomms/fcz047 PMC7100005

[131] Zhang Q, Sidorenko J, Couvy-Duchesne B, et al Risk prediction of late-onset Alzheimer’s disease implies an oligogenic architecture. Nat Commun. 2020;11(1):4799. 32968074 10.1038/s41467-020-18534-1 PMC7511365

[132] Stevenson-Hoare J, Heslegrave A, Leonenko G, et al Plasma biomarkers and genetics in the diagnosis and prediction of Alzheimer’s disease. Brain. 2023;146(2):690–699. 35383826 10.1093/brain/awac128 PMC9924904

[133] Rasmussen KL, Tybjærg-Hansen A, Nordestgaard BG, Frikke-Schmidt R. Absolute 10-year risk of dementia by age, sex and *APOE* genotype: A population-based cohort study. CMAJ. 2018;190(35):E1033–E1041. 30181149 10.1503/cmaj.180066 PMC6148649

[134] Yengo L, Vedantam S, Marouli E, et al A saturated map of common genetic variants associated with human height. Nature. 2022;610(7933):704–712. 36224396 10.1038/s41586-022-05275-y PMC9605867

[135] Jansen IE, van der Lee SJ, Gomez-Fonseca D, et al Genome-wide meta-analysis for Alzheimer’s disease cerebrospinal fluid biomarkers. Acta Neuropathol. 2022;144(5):821–842. 36066633 10.1007/s00401-022-02454-z PMC9547780

[136] Davies G, Lam M, Harris SE, et al Study of 300,486 individuals identifies 148 independent genetic loci influencing general cognitive function. Nat Commun. 2018;9(1):2098. 29844566 10.1038/s41467-018-04362-x PMC5974083

[137] Paliwal D, McInerney TW, Pa J, et al Mitochondrial pathway polygenic risk scores are associated with Alzheimer’s disease. Neurobiol Aging. 2021;108:213–222. 34521561 10.1016/j.neurobiolaging.2021.08.005

[138] Harrison JR, Foley SF, Baker E, et al Pathway-specific polygenic scores for Alzheimer’s disease are associated with changes in brain structure in younger and older adults. Brain Commun. 2023;5(5):fcad229. 37744023 10.1093/braincomms/fcad229 PMC10517196

[139] Darst BF, Koscik RL, Racine AM, et al Pathway-specific polygenic risk scores as predictors of amyloid-β deposition and cognitive function in a sample at increased risk for Alzheimer’s disease. J Alzheimers Dis. 2017;55(2):473–484. 27662287 10.3233/JAD-160195 PMC5123972

[140] Hernández CF, Villaman C, Leu C, et al Polygenic score analysis identifies distinct genetic risk profiles in Alzheimer’s disease comorbidities. Sci Rep. 2025;15(1):11407. 40181078 10.1038/s41598-025-95755-8 PMC11968852

[141] Schork NJ, Elman JA; Alzheimer’s Disease Neuroimaging Initiative. Pathway-specific polygenic risk scores correlate with clinical Status and Alzheimer’s disease-related biomarkers. J Alzheimers Dis. 2023;95:915–929. 37661888 10.3233/JAD-230548 PMC10697039

[142] Sinnott-Armstrong N, Tanigawa Y, Amar D, et al Genetics of 35 blood and urine biomarkers in the UK biobank. Nat Genet. 2021;53(2):185–194. 33462484 10.1038/s41588-020-00757-z PMC7867639

[143] Amariuta T, Ishigaki K, Sugishita H, et al Improving the trans-ancestry portability of polygenic risk scores by prioritizing variants in predicted cell-type-specific regulatory elements. Nat Genet. 2020;52(12):1346–1354. 33257898 10.1038/s41588-020-00740-8 PMC8049522

[144] Escott-Price V, Schmidt KM. Probability of Alzheimer’s disease based on common and rare genetic variants. Alzheimers Res Ther. 2021;13(1):140. 34404470 10.1186/s13195-021-00884-7 PMC8369699

[145] Nicolas A, Sherva R, Grenier-Boley B, et al Transferability of European-derived Alzheimer’s disease polygenic risk scores across multiancestry populations. Nat Genet. 2025;57(7):1598–1610. 40533518 10.1038/s41588-025-02227-w PMC12283339

[146] Tan CH, Bonham LW, Fan CC, et al Polygenic hazard score, amyloid deposition and Alzheimer’s neurodegeneration. Brain. 2019;142(2):460–470. 30689776 10.1093/brain/awy327 PMC6351776

[147] Farhadieh ME, Mozafar M, Sanaaee S, et al Polygenic hazard score predicts synaptic and axonal degeneration and cognitive decline in Alzheimer’s disease continuum. Arch Gerontol Geriatr. 2024;127:105576. 39096557 10.1016/j.archger.2024.105576

[148] Kauppi K, Fan CC, McEvoy LK, et al Combining polygenic hazard score with volumetric MRI and cognitive measures improves prediction of progression from mild cognitive impairment to Alzheimer’s disease. Front Neurosci. 2018;12:348529. 10.3389/fnins.2018.00260 PMC5937163 29760643

[149] Banks SJ, Qiu Y, Fan CC, et al Enriching the design of Alzheimer’s disease clinical trials: Application of the polygenic hazard score and composite outcome measures. Alzheimers Dement (N Y). 2020;6(1):e12071. 32999917 10.1002/trc2.12071 PMC7507583

[150] Maloney JA, Bainbridge T, Gustafson A, et al Molecular mechanisms of Alzheimer disease protection by the A673T allele of amyloid precursor protein. J Biol Chem. 2014;289(45):30990–31000. 25253696 10.1074/jbc.M114.589069 PMC4223305

[151] Reiman EM, Arboleda-Velasquez JF, Quiroz YT, et al Exceptionally low likelihood of Alzheimer’s dementia in APOE2 homozygotes from a 5,000-person neuropathological study. Nat Commun. 2020;11(1):667. 32015339 10.1038/s41467-019-14279-8 PMC6997393

[152] Arboleda-Velasquez JF, Lopera F, O’Hare M, et al Resistance to autosomal dominant Alzheimer’s disease in an APOE3 Christchurch homozygote: A case report. Nat Med. 2019;25(11):1680–1683. 31686034 10.1038/s41591-019-0611-3 PMC6898984

[153] Protective genetic variants against Alzheimer’s disease. Lancet Neurol. 2025;24(6):524–534. 40409316 10.1016/S1474-4422(25)00116-4 PMC12614636

[154] Belloy ME, Napolioni V, Han SS, Le Guen Y, Greicius MD. Association of klotho-VS heterozygosity with risk of Alzheimer disease in individuals who carry APOE4. JAMA Neurol. 2020;77(7):849–862. 32282020 10.1001/jamaneurol.2020.0414 PMC7154955

[155] Fehér Á, Giricz Z, Juhász A, Pákáski M, Janka Z, Kálmán J. ABCA1 rs2230805 and rs2230806 common gene variants are associated with Alzheimer’s disease. Neurosci Lett. 2018;664:79–83. 29133174 10.1016/j.neulet.2017.11.027

[156] Sassi C, Nalls MA, Ridge PG, et al ABCA7 p.G215S as potential protective factor for Alzheimer’s disease. Neurobiol Aging. 2016;46:235.e1–235.e9. 10.1016/j.neurobiolaging.2016.04.004 PMC5024078 27289440

[157] Zhang CC, Wang HF, Tan MS, et al SORL1 is associated with the risk of late-onset Alzheimer’s disease: A replication study and meta-analyses. Mol Neurobiol. 2017;54(3):1725–1732. 26873856 10.1007/s12035-016-9780-y

[158] Ridge PG, Karch CM, Hsu S, et al Linkage, whole genome sequence, and biological data implicate variants in RAB10 in Alzheimer’s disease resilience. Genome Med. 2017;9(1):100. 29183403 10.1186/s13073-017-0486-1 PMC5706401

[159] Santos-Rebouças CB, Gonçalves AP, Dos Santos JM, et al Rs3851179 polymorphism at 5’ to the PICALM gene is associated with Alzheimer and Parkinson diseases in Brazilian population. Neuromolecular Med. 2017;19(2-3):293–299. 28567584 10.1007/s12017-017-8444-z

[160] Masri I, Salami A, El Shamieh S, Bissar-Tadmouri N. Rs3851179g>A in PICALM is protective against Alzheimer’s disease in five different countries surrounding the Mediterranean. Curr Aging Sci. 2020;13(2):162–168. 31648652 10.2174/1874609812666191019143237

[161] Benitez BA, Jin SC, Guerreiro R, et al Missense variant in TREML2 protects against Alzheimer’s disease. Neurobiol Aging. 2014;35(6):1510.e19–1510.e26. 10.1016/j.neurobiolaging.2013.12.010 PMC3961557 24439484

[162] Staley HA, Jernigan JE, Bolen ML, et al Alzheimer’s disease-associated protective variant Plcg2-P522R modulates peripheral macrophage function in a sex-dimorphic manner. J Neuroinflammation. 2024;21(1):280. 39487477 10.1186/s12974-024-03271-9 PMC11529260

[163] Lopera F, Marino C, Chandrahas AS, et al Resilience to autosomal dominant Alzheimer’s disease in a reelin-COLBOS heterozygous man. Nat Med. 2023;29(5):1243–1252. 37188781 10.1038/s41591-023-02318-3 PMC10202812

[164] Hou J, Hess JL, Armstrong N, et al Polygenic resilience scores capture protective genetic effects for Alzheimer’s disease. Transl Psychiatry. 2022;12(1):296. 35879306 10.1038/s41398-022-02055-0 PMC9314356

[165] Naslavsky MS, Suemoto CK, Brito LA, et al Global and local ancestry modulate APOE association with Alzheimer’s neuropathology and cognitive outcomes in an admixed sample. Mol Psychiatry. 2022;27(11):4800–4808. 36071110 10.1038/s41380-022-01729-x PMC9734036

[166] NIAGADS: The National Institute on Aging Genetics of Alzheimer’s Disease Data Storage Site . Accessed 14 August 2025. https://www.niagads.org/

[167] Kuksa PP, Liu CL, Fu W, et al Alzheimer’s disease variant portal: A catalog of genetic findings for Alzheimer's disease. J Alzheimers Dis. 2022;86(1):461–477. 35068457 10.3233/JAD-215055 PMC9028687

[168] Alzheimer Europe . Research. Accessed 14 August 2025. https://www.alzheimer-europe.org/research?language_content_entity=en.

[169] Sims R, Hill M, Williams J. The multiplex model of the genetics of Alzheimer’s disease. Nat Neurosci. 2020;23(3):311–322. 32112059 10.1038/s41593-020-0599-5

[170] Gouveia Roque C, Phatnani H, Hengst U. The broken Alzheimer’s disease genome. Cell Genom. 2024;4(5):100555. 38697121 10.1016/j.xgen.2024.100555 PMC11099344

[171] Stephens MC, Li J, Mair M, et al Computational and functional prioritization identifies genes that rescue behavior and reduce tau protein in fly and human cell models of Alzheimer disease. Am J Hum Genet. 2025;112(5):1081–1096. 40215969 10.1016/j.ajhg.2025.03.012 PMC12120185

[172] Zhong H, Zhu J, Liu S, et al Linking DNA methylation in brain regions to Alzheimer’s disease risk: A Mendelian randomization study. Hum Mol Genet. 2025;34(12):1026–1033. 40267236 10.1093/hmg/ddaf053 PMC13377460

[173] Yarbro JM, Shrestha HK, Wang Z, et al Proteomic landscape of Alzheimer’s disease: Emerging technologies, advances and insights (2021–2025). Mol Neurodegener. 2025;20(1):83. 40660303 10.1186/s13024-025-00874-5 PMC12257826

[174] Bai B, Wang X, Li Y, et al Deep multilayer brain proteomics identifies molecular networks in Alzheimer’s disease progression. Neuron. 2020;106(4):700. 32437656 10.1016/j.neuron.2020.04.031 PMC7322979

[175] Griffiths J, Schneegans E, Whitwell H, et al A synaptic-astrocytic proteomic signature associated with synaptopathy in Alzheimer’s Disease. bioRxiv. [Preprint] doi:10.1101/2025.01.23.634408

[176] Weissman L, Jo DG, Sørensen MM, et al Defective DNA base excision repair in brain from individuals with Alzheimer’s disease and amnestic mild cognitive impairment. Nucleic Acids Res. 2007;35(16):5545–5555. 17704129 10.1093/nar/gkm605 PMC2018628

[177] Liu PP, Xie Y, Meng XY, Kang JS. History and progress of hypotheses and clinical trials for Alzheimer’s disease. Signal Transduct Target Ther. 2019;4:29. 31637009 10.1038/s41392-019-0063-8 PMC6799833

[178] Bracher-Smith M, Melograna F, Ulm B, et al Machine learning in Alzheimer’s disease genetics. Nat Commun. 2025;16(1):6726. 40691194 10.1038/s41467-025-61650-z PMC12280214

[179] Avsec Ž, Latysheva N, Cheng J, et al Advancing regulatory variant effect prediction with AlphaGenome. Nature. 2026;649:1206–1218. 41606153 10.1038/s41586-025-10014-0 PMC12851941

[180] Nguyen E, Poli M, Faizi M, et al HyenaDNA: long-range genomic sequence modeling at single nucleotide resolution. Adv Neural Inf Process Syst. 2023:36.

[181] Nath A, Mwesigwa S, Dai Y, Jiang X, Zhao Z. GENEVIC: GENetic data exploration and visualization via intelligent interactive console. Bioinformatics. 2024;40(10):btae500. 39115390 10.1093/bioinformatics/btae500 PMC11467054

[182] Lin A, Ye J, Qi C, et al Bridging artificial intelligence and biological sciences: A comprehensive review of large language models in bioinformatics. Brief Bioinform. 2025;26(4):bbaf357. 40708223 10.1093/bib/bbaf357 PMC12289552

