# Supplementary material for: Alzheimer’s disease: genetic background in the era of next-generation sequencing technologies
Source: Brain Commun. 2026 Sep 16;8(5):fcag332. doi: 10.1093/braincomms/fcag332 (PMC13579704; doi:10.1093/braincomms/fcag332)
Supplement: Supplementary file 1 [file fcag332_supplementary_data.docx]

## **Supplementary Table 1: Selected Copy number variants (CNVs) in Alzheimer's disease and their functional impact.**

| **Gene/Region** | **Location** | **CNV Type** | **Functional Impact** | **Reference** |
| --- | --- | --- | --- | --- |
| *APP* | 21q21.3 | Gene duplication | ↑ APP dosage → ↑ Aβ accumulation; early-onset AD | [^1^](https://paperpile.com/c/QhS7B4/jb0rX) |
| *PSEN1* | 14q24.2 | Exonic deletion | Disrupted APP processing; familial AD | [^2,3^](https://paperpile.com/c/QhS7B4/sVTpp+rD39G) |
| *CR1* | 1q32.2 | Duplication | ↑ Complement activation; immune dysregulation | [^4,5^](https://paperpile.com/c/QhS7B4/Q6GG8+iStwq) |
| *CHRFAM7A/ CHRNA7* | 15q13.2/ 15q13.3 | Duplication/ Deletion | Cholinergic signaling modulation; ↑ or ↓ AD risk depending on direction | [^6–8^](https://paperpile.com/c/QhS7B4/qHP4R+G069z+P1GsC) |
| *CREB1/FAM119A* | 2q33.3 | Deletion | ↓ CREB1, FAM119A expression → memory impairment | [^9^](https://paperpile.com/c/QhS7B4/tlVdk) |
| *ABCA7/ABCA1* | 19p13.3 | Deletion | Impaired lipid transport and microglial function | [^10^](https://paperpile.com/c/QhS7B4/BIZC6) |
| *CTSB* | 8p23.1 | Deletion | Lysosomal dysfunction, impaired protein degradation | [^10^](https://paperpile.com/c/QhS7B4/BIZC6) |
| *22q11.21* | 22q11.21 | Deletion | Overlaps DiGeorge region; immune/neurodevelopmental dysfunction | [^10^](https://paperpile.com/c/QhS7B4/BIZC6) |
| *APC2* | 19p13.3 | Duplication | ↓ Risk of psychosis in AD | [^11^](https://paperpile.com/c/QhS7B4/yRdWI) |
| *CPNE4* | 3q22.1 | Deletion | Intragenic deletion, synaptic signaling | [^12^](https://paperpile.com/c/QhS7B4/8nA9I) |
| *CYFIP1* | 15q11.2 | Gene duplication | Synaptic function; cognitive variability | [^13^](https://paperpile.com/c/QhS7B4/S98J5) |
| *SULT1A3/4* | 16p11.2 | Duplication | Detoxification/metabolic processes | [^14^](https://paperpile.com/c/QhS7B4/BBAYX) |
| *AMY1A* | 1p21.1 | Duplication | Amylase dosage, unclear role in AD | [^15^](https://paperpile.com/c/QhS7B4/GL8ey) |
| *RELN/DOPEY2/SET* | Various | CNVs | Synaptic plasticity and neurodevelopmental disruption | [^6^](https://paperpile.com/c/QhS7B4/qHP4R) |
| *HLA-DRA/HLA-DPB1* | Various | CNVs | Altered MHC II signaling; neuroinflammation | [^6^](https://paperpile.com/c/QhS7B4/qHP4R) |
| *MAGI1, NLRP8, KLK6, MEOX2, SLC30A3, FPR2, SVOP* | Various | Duplication | Early-onset AD; cell adhesion, inflammation | [^1^](https://paperpile.com/c/QhS7B4/jb0rX) |
| *DEL5006/mCNV14192/DUP42180* | Various | Deletion/Duplication | Longevity, plasminogen activation, immune response modulation | [^16^](https://paperpile.com/c/QhS7B4/o1W6K) |
| *NRXN1/ERBB4/IMMP2L* | Various | CNVs | Disrupted synaptic or mitochondrial function | [^6^](https://paperpile.com/c/QhS7B4/qHP4R) |
| *CSMD1, HNRNPCL1, SLC35F2* | Various | CNV | ↑ CNV burden in AD cases | [^7^](https://paperpile.com/c/QhS7B4/G069z) |
| *SET, JAG2, ZFPM1* | Various | Duplication | Suggested link to psychosis in AD | [^11^](https://paperpile.com/c/QhS7B4/yRdWI) |
| *A2BP1, ABAT, CDH2,*  *CRMP1, DMRT1, EPHA5,*  *EPHA6, ERMP1, EVC,*  *EVC2, FLJ35024, VLDLR* | Various | CNVs | Rare, non-recurrent CNVs; possible roles  in neuronal projection and adhesion | [^17^](https://paperpile.com/c/QhS7B4/fV23Z) |

AD - Alzheimer’s disease; Aβ - amyloid beta; CNVs - Copy number variations; MHC II - Major Histocompatibility Complex Class II

**Supplementary Table 1 references**

1.	[Rovelet-Lecrux A, Hannequin D, Raux G, et al. APP locus duplication causes autosomal dominant early-onset Alzheimer disease with cerebral amyloid angiopathy. *Nat Genet*. 2006;38(1):24-26.](http://paperpile.com/b/QhS7B4/jb0rX)

2.	[Le Guennec K, Veugelen S, Quenez O, et al. Deletion of exons 9 and 10 of the Presenilin 1 gene in a patient with Early-onset Alzheimer Disease generates longer amyloid seeds. *Neurobiol Dis*. 2017;104:97-103.](http://paperpile.com/b/QhS7B4/sVTpp)

3.	[Crook R, Verkkoniemi A, Perez-Tur J, et al. A variant of Alzheimer’s disease with spastic paraparesis and unusual plaques due to deletion of exon 9 of presenilin 1. *Nat Med*. 1998;4(4):452-455.](http://paperpile.com/b/QhS7B4/rD39G)

4.	[Brouwers N, Van Cauwenberghe C, Engelborghs S, et al. Alzheimer risk associated with a copy number variation in the complement receptor 1 increasing C3b/C4b binding sites. *Mol Psychiatry*. 2012;17(2):223-233.](http://paperpile.com/b/QhS7B4/Q6GG8)

5.	[Lambert JC, Heath S, Even G, et al. Genome-wide association study identifies variants at CLU and CR1 associated with Alzheimer’s disease. *Nat Genet*. 2009;41(10):1094-1099.](http://paperpile.com/b/QhS7B4/iStwq)

6.	[Swaminathan S, Shen L, Kim S, et al. Analysis of copy number variation in Alzheimer’s disease: the NIALOAD/ NCRAD Family Study. *Curr Alzheimer Res*. 2012;9(7):801-814.](http://paperpile.com/b/QhS7B4/qHP4R)

7.	[Swaminathan S, Kim S, Shen L, et al. Genomic Copy Number Analysis in Alzheimer’s Disease and Mild Cognitive Impairment: An ADNI Study. *Int J Alzheimers Dis*. 2011;2011:729478.](http://paperpile.com/b/QhS7B4/G069z)

8.	[Heinzen EL, Need AC, Hayden KM, et al. Genome-wide scan of copy number variation in late-onset Alzheimer’s disease. *J Alzheimers Dis*. 2010;19(1):69-77.](http://paperpile.com/b/QhS7B4/P1GsC)

9.	[Li Y, Shaw CA, Sheffer I, et al. Integrated copy number and gene expression analysis detects a CREB1 association with Alzheimer’s disease. *Transl Psychiatry*. 2012;2(11):e192.](http://paperpile.com/b/QhS7B4/tlVdk)

10.	[Quenez O, Schramm C, Cassinari K, et al. Extremely rare CNVs contributing to Alzheimer disease risk: a case-control association analysis of exome sequencing data from 22,319 individuals. *medRxiv*. Published online November 1, 2024. doi:](http://paperpile.com/b/QhS7B4/BIZC6)[10.1101/2024.10.28.24314051](http://dx.doi.org/10.1101/2024.10.28.24314051)

11.	[Zheng X, Demirci FY, Barmada MM, et al. Genome-wide copy-number variation study of psychosis in Alzheimer’s disease. *Transl Psychiatry*. 2015;5(6):e574.](http://paperpile.com/b/QhS7B4/yRdWI)

12.	[Szigeti K, Lal D, Li Y, et al. Genome-wide scan for copy number variation association with age at onset of Alzheimer’s disease. *J Alzheimers Dis*. 2013;33(2):517-523.](http://paperpile.com/b/QhS7B4/8nA9I)

13.	[Ghani M, Pinto D, Lee JH, et al. Genome-wide survey of large rare copy number variants in Alzheimer’s disease among Caribbean hispanics. *G3 (Bethesda)*. 2012;2(1):71-78.](http://paperpile.com/b/QhS7B4/S98J5)

14.	[Butcher NJ, Horne MK, Mellick GD, et al. Sulfotransferase 1A3/4 copy number variation is associated with neurodegenerative disease. *Pharmacogenomics J*. 2018;18(2):209-214.](http://paperpile.com/b/QhS7B4/BBAYX)

15.	[Byman E, Nägga K, Gustavsson AM, et al. Alpha-amylase 1A copy number variants and the association with memory performance and Alzheimer’s dementia. *Alzheimers Res Ther*. 2020;12(1):158.](http://paperpile.com/b/QhS7B4/GL8ey)

16.	[Hao Y, Li C, Wang H, Ming C. Effects of copy number variations on longevity in late-onset Alzheimer’s disease patients: insights from a causality network analysis. *Front Aging Neurosci*. 2023;15:1241412.](http://paperpile.com/b/QhS7B4/o1W6K)

17.	[Hooli BV, Kovacs-Vajna ZM, Mullin K, et al. Rare autosomal copy number variations in early-onset familial Alzheimer’s disease. *Mol Psychiatry*. 2014;19(6):676-681.](http://paperpile.com/b/QhS7B4/fV23Z)

**Supplementary Table 2: Selected genetic variants in Alzheimer’s disease divided by functional category.**

| **Functional Category** | **Genes / Loci *** | **SNPs (Examples)** | **CNVs (Gene/Region)** | **STRs / TRs** | **TEs (Gene/Region)** |
| --- | --- | --- | --- | --- | --- |
| Neurotransmission * | *BIN1, CD2AP, CLU, PTK2B, SHARPIN, PICALM, APP, ICA1, GRN, ADAM10, SNX1, DOC2A, RABEP1, TSPOAP1, APOE, ANK3, FERMT2, PKP4, CCNY, GRID2* | *BIN1* (rs744373)[^1,2^](https://paperpile.com/c/rYOPiV/1MlX+Q2np), *CLU* (rs11136000)[^3^](https://paperpile.com/c/rYOPiV/4qZWN), *PICALM* (rs3851179)[^4^](https://paperpile.com/c/rYOPiV/6i7rJ), *PTK2B* (rs28834970)[^5^](https://paperpile.com/c/rYOPiV/kc2zy) | *NRXN1* intronic CNV, *GRID2* deletion[^6^](https://paperpile.com/c/rYOPiV/H85eE) | *GRID2* STR expansion[^7^](https://paperpile.com/c/rYOPiV/KJju3), *APOE* STR[^3,7–9^](https://paperpile.com/c/rYOPiV/P1vGg+4qZWN+GcPmP+KJju3),  *MINDY*/*ADAM10*[^8^](https://paperpile.com/c/rYOPiV/P1vGg), *CLU*[^8^](https://paperpile.com/c/rYOPiV/P1vGg), *BIN*[^8^](https://paperpile.com/c/rYOPiV/P1vGg), *CCNY*[^10^](https://paperpile.com/c/rYOPiV/JsDFZ) | – |
| Immune response* | *CR1, ADAM17, INPP5D, CLNK, RHOH, TNIP1, TREM2, BLNK, ABCA7, APP, GRN, HLA, SEC61G, EPHA1, CTSB, CLU, PTB2B, SHARPIN, SIGLEC11, NCK2, ABI3, SPI1, MS4A, ADAM10, CTSH, IL34, PLCG2, SCIMP, LILRB2, MME, TSPAN14, RBCK1, MUC6, DSC3* | *TREM2* (rs75932628)[^11,12^](https://paperpile.com/c/rYOPiV/RWhXK+kcjSl), *CR1* (rs6656401)[^13^](https://paperpile.com/c/rYOPiV/CYYTY), *CD33* (rs3865444)[^14^](https://paperpile.com/c/rYOPiV/Pf2Ng), *MS4A4A* (rs1582763)[^15^](https://paperpile.com/c/rYOPiV/XhSjT) | LCR-associated CNV in *CR1*[^16^](https://paperpile.com/c/rYOPiV/ctDPc), *CHRFAM7A* exon deletion[^16,17^](https://paperpile.com/c/rYOPiV/ctDPc+XKCmG) | *HLA-DRB1* STR[^8^](https://paperpile.com/c/rYOPiV/P1vGg), *ABCA7*[^8^](https://paperpile.com/c/rYOPiV/P1vGg), *CR1*[^8^](https://paperpile.com/c/rYOPiV/P1vGg), *MUC6*[^18^](https://paperpile.com/c/rYOPiV/UdzQX), *DSC3*[^10^](https://paperpile.com/c/rYOPiV/JsDFZ) | SINE insertion at *C1QTNF4*[^19^](https://paperpile.com/c/rYOPiV/Mk9ga) |
| Endo-lysosomal network* | *SORT1, BIN1, CD2AP, TMEM106B, CTSB, PICALM, SORL1, TPCN1, RIN3, SPPL2A, SNX1, CTSH, WDR81, GRN, RABEP1, APOE* | *TMEM106B* (rs1990622), *SORL1* (rs2298813), *CD2AP* (rs10948363) | *CTSB* deletion[^20^](https://paperpile.com/c/rYOPiV/4XV9C) | *APOE* STR[^7,8^](https://paperpile.com/c/rYOPiV/P1vGg+KJju3), BIN1[^8^](https://paperpile.com/c/rYOPiV/P1vGg) | – |
| APP processing & clearance* | *ADAM17, BIN1, MME, CTSB, CLU, PTK2B, ABCA1, PICALM, SORL1, ADAM10, APH1B, SNX1, CD2AP, TSPAN14, LILRB2, SORT1, TREM2, ABCA7, APOE, APP, GRAP* | *APP* (V717I)[^21^](https://paperpile.com/c/rYOPiV/9wY2V), *PSEN1* (rs63750082)[^13^](https://paperpile.com/c/rYOPiV/CYYTY), *SORL1* (rs11218343)[^22^](https://paperpile.com/c/rYOPiV/D1d0X) | *APP* duplication (chr21q21.3)[^23^](https://paperpile.com/c/rYOPiV/zc4cv), *PSEN1* exon 9 and 10 del[^24^](https://paperpile.com/c/rYOPiV/3qWTd) | *APOE* STR[^7,8^](https://paperpile.com/c/rYOPiV/P1vGg+KJju3), *ABCA7*[^8^](https://paperpile.com/c/rYOPiV/P1vGg), *BIN1*[^8^](https://paperpile.com/c/rYOPiV/P1vGg), *PICALM*[^8^](https://paperpile.com/c/rYOPiV/P1vGg), *MINDY*/*ADAM10*[^8^](https://paperpile.com/c/rYOPiV/P1vGg), *GRAP*[^10^](https://paperpile.com/c/rYOPiV/JsDFZ) | Alu insertion in *APP*[^25^](https://paperpile.com/c/rYOPiV/TKMHT) |
| Tau* | *CLU, PTK2B, SORL1, MAPT, APOE, TREM2, CD2AP, PICALM, MAP6* | *MAPT* (rs242557)[^26^](https://paperpile.com/c/rYOPiV/d6qtm), *BIN1* (rs744373)[^2,3^](https://paperpile.com/c/rYOPiV/4qZWN+Q2np) | *MAPT* duplication[^27^](https://paperpile.com/c/rYOPiV/nplNS) | *APOE* STR[^7,8^](https://paperpile.com/c/rYOPiV/P1vGg+KJju3), *MAP6*[^10^](https://paperpile.com/c/rYOPiV/JsDFZ) | SVA deletion near *BIN1*[^28^](https://paperpile.com/c/rYOPiV/s2AFJ) |
| Lipid metabolism* | *SORT1, TREM2, ABCA1, ECHDC3, ABCA7, APOE, JAZF1* | *APOE* ε4  (rs429358)[^29^](https://paperpile.com/c/rYOPiV/LIXts), *ABCA7* (rs3764650)[^30^](https://paperpile.com/c/rYOPiV/wKBgd), *ABCA1* (rs2230806)[^31^](https://paperpile.com/c/rYOPiV/1h8Js) | *ABCA7* exon 18–19 del, *ABCA1* exonic del [^20^](https://paperpile.com/c/rYOPiV/4XV9C) | *ABCA7* VNTR[^32,33^](https://paperpile.com/c/rYOPiV/vVjMa+dR8xG), *ABCA7* STR[^8^](https://paperpile.com/c/rYOPiV/P1vGg),  *APOE* STR[^7,8^](https://paperpile.com/c/rYOPiV/P1vGg+KJju3) | – |
| Gene expression* | *KAT8, MAF, PRDM7, SPI1, FOXF1, KLF16, WDR12, JAZF1, ZCWPW1, CELF1, SLC24A4* | *ZCWPW1* (rs1476679)[^34^](https://paperpile.com/c/rYOPiV/Hvkat), *CELF1* (rs10838725)[^35^](https://paperpile.com/c/rYOPiV/kRkrS) | – | – | – |
| Heparan sulfate metabolism* | *IDUA, HS3ST1, HS3ST5* | – | – | – | – |
| TNFα signaling* | *SPPL2A, SHARPIN, ADAM17, TNIP1, RBCK1* | – | – | – | – |
| Neurodevelopment | *RELN, DOPEY2, SET, ZFPM1, CNTLN, IMMP2L*  *SNX32* | *RELN* (rs362771)[^36^](https://paperpile.com/c/rYOPiV/0UgY5) | *SET* duplication, *CNTLN* deletion[^37^](https://paperpile.com/c/rYOPiV/Bdmxw), *RELN* CNV, *IMMP2L* deletion[^6^](https://paperpile.com/c/rYOPiV/H85eE) | *RELN* STRs (5′ UTR, intronic)[^38^](https://paperpile.com/c/rYOPiV/mpA9),  *SNX32*[^8^](https://paperpile.com/c/rYOPiV/P1vGg) | – |
| Cerebrovascular Function | *PLAU, PLGRKT, CALB2* | *PLAU* (rs2227564)[^39^](https://paperpile.com/c/rYOPiV/KVRM8) | CNVs (DEL5006, DUP42180) regulating *PLAU*, *PLGRKT*, *CALB2*[^40^](https://paperpile.com/c/rYOPiV/80C8W) |  | – |
| Detoxification / Oxidative Stress | *GSTT1, ADI1, CTSB, WBS1* |  | *CTSB* deletion (exonic)[^20^](https://paperpile.com/c/rYOPiV/4XV9C), *GSTT1* duplication[^6^](https://paperpile.com/c/rYOPiV/H85eE) | *WBS1*[^8^](https://paperpile.com/c/rYOPiV/P1vGg) | – |
| Mitochondrial Import / APOE linkage | *TOMM40* | *TOMM40* rs2075650[^41^](https://paperpile.com/c/rYOPiV/qEdMn) | – | TOMM40 rs10524523 poly-T STR (linked to *APOE* ε haplotypes)[^42^](https://paperpile.com/c/rYOPiV/CCY34) | Alu insertion in TOMM40 intron 6[^43^](https://paperpile.com/c/rYOPiV/3p5R7) |
| Psychiatric / Cognitive Modifiers | *APC2, SET, JAG2, ZFPM1, CNTLN* | – | *APC2* duplication (protective), *CNTLN* deletion (risk)[^37^](https://paperpile.com/c/rYOPiV/Bdmxw) | – | – |

The first 9 categories (marked with *) and corresponding genes/loci are inspired by Fig. 1 in [^100^](https://paperpile.com/c/GHQICl/T1nQ5). We list the main variants mentioned in the text. In the case of STRs, it seems that rather than individual TR expansions, the high burden of STR expansions in the genome increases the risk of Alzheimer’s disease, underscoring the pivotal role of genomic instability in the onset and progression of Alzheimer’s disease. However, for illustration we mention some STRs pinpointed in the cited literature and mentioned in the text. More CNVs involved in Alzheimer’s disease can be found in **Supplementary Table 1**. CNVs - Copy number variations, del - single or more nucleotides deletion, rs - reference SNP cluster identifier (also called rsID) for SNP variants, SINEs - Short interspersed nuclear elements, SNPs - Single nucleotide polymorphisms, STRs - Short tandem repeats, TEs - Transposable elements, TRs - Tandem repeats

**Supplementary Table 2 references**

1.	[Liu, G. *et al.* The CLU gene rs11136000 variant is significantly associated with Alzheimer’s disease in Caucasian and Asian populations. *Neuromolecular Med* 16, 52–60 (2014).](http://paperpile.com/b/rYOPiV/1MlX)

2.	[Voskobiynyk, Y. *et al.* Alzheimer’s disease risk gene induces Tau-dependent network hyperexcitability. *Elife* 9, (2020).](http://paperpile.com/b/rYOPiV/Q2np)

3.	[Liu, G. *et al.* The CLU gene rs11136000 variant is significantly associated with Alzheimer’s disease in Caucasian and Asian populations. *Neuromolecular Med* 16, 52–60 (2014).](http://paperpile.com/b/rYOPiV/4qZWN)

4.	[Kamboh, M. I. *et al.* Genome-wide association analysis of age-at-onset in Alzheimer’s disease. *Mol Psychiatry* 17, 1340–1346 (2012).](http://paperpile.com/b/rYOPiV/6i7rJ)

5.	[Sakae, N. *et al.* Evaluation of Associations of Alzheimer’s Disease Risk Variants that Are Highly Expressed in Microglia with Neuropathological Outcome Measures. *J Alzheimers Dis* 70, 659–666 (2019).](http://paperpile.com/b/rYOPiV/kc2zy)

6.	[Swaminathan, S. *et al.* Analysis of copy number variation in Alzheimer’s disease: the NIALOAD/ NCRAD Family Study. *Curr Alzheimer Res* 9, 801–814 (2012).](http://paperpile.com/b/rYOPiV/H85eE)

7.	[Guo, M. H., Lee, W.-P., Vardarajan, B., Schellenberg, G. D. & Phillips-Cremins, J. E. Polygenic burden of short tandem repeat expansions promotes risk for Alzheimer’s disease. *Nat. Commun.* 16, 1126 (2025).](http://paperpile.com/b/rYOPiV/KJju3)

8.	[Gmelin, D. *et al.* GWAS on short tandem repeats identifies novel genetic mechanisms in Alzheimer’s disease. *medRxiv* (2025) doi:10.1101/2025.03.13.25323833.](http://paperpile.com/b/rYOPiV/P1vGg)

9.	[Voskobiynyk, Y. *et al.* Alzheimer’s disease risk gene induces Tau-dependent network hyperexcitability. *Elife* 9, (2020).](http://paperpile.com/b/rYOPiV/GcPmP)

10.	[Heath, A., McNerney, M. W. & Yesavage, J. Whole Genome Variable Number Tandem Repeat Analysis in Alzheimer Disease. *Neurol Genet* 11, e200241 (2025).](http://paperpile.com/b/rYOPiV/JsDFZ)

11.	[Kiianitsa, K. *et al.* TREM2 variants that cause early dementia and increase Alzheimer’s disease risk affect gene splicing. *Brain* 147, 2368–2383 (2024).](http://paperpile.com/b/rYOPiV/RWhXK)

12.	[Gratuze, M., Leyns, C. E. G. & Holtzman, D. M. New insights into the role of TREM2 in Alzheimer’s disease. *Molecular Neurodegeneration* 13, 1–16 (2018).](http://paperpile.com/b/rYOPiV/kcjSl)

13.	[Krishnamurthy, H. K. *et al.* An overview of the genes and biomarkers in Alzheimer’s disease. *Ageing Res Rev* 104, 102599 (2025).](http://paperpile.com/b/rYOPiV/CYYTY)

14.	[Bissar, N., Kassir, R., Salami, A. & El Shamieh, S. Association of immunity-related gene SNPs with Alzheimer’s disease. *Exp. Biol. Med.* 249, 10303 (2024).](http://paperpile.com/b/rYOPiV/Pf2Ng)

15.	[Deming, Y. *et al.* The gene cluster is a key modulator of soluble TREM2 and Alzheimer’s disease risk. *Sci Transl Med* 11, (2019).](http://paperpile.com/b/rYOPiV/XhSjT)

16.	[Brouwers, N. *et al.* Alzheimer risk associated with a copy number variation in the complement receptor 1 increasing C3b/C4b binding sites. *Mol Psychiatry* 17, 223–233 (2012).](http://paperpile.com/b/rYOPiV/ctDPc)

17.	[Swaminathan, S. *et al.* Genomic Copy Number Analysis in Alzheimer’s Disease and Mild Cognitive Impairment: An ADNI Study. *Int J Alzheimers Dis* 2011, 729478 (2011).](http://paperpile.com/b/rYOPiV/XKCmG)

18.	[Katsumata, Y. *et al.* Alzheimer Disease Pathology-Associated Polymorphism in a Complex Variable Number of Tandem Repeat Region Within the MUC6 Gene, Near the AP2A2 Gene. *J Neuropathol Exp Neurol* 79, 3–21 (2020).](http://paperpile.com/b/rYOPiV/UdzQX)

19.	[Feng, Y. *et al.* Widespread transposable element dysregulation in human aging brains with Alzheimer’s disease. *Alzheimers Dement* 20, 7495–7517 (2024).](http://paperpile.com/b/rYOPiV/Mk9ga)

20.	[Quenez, O. *et al.* Extremely rare CNVs contributing to Alzheimer disease risk: a case-control association analysis of exome sequencing data from 22,319 individuals. *medRxiv* (2024) doi:10.1101/2024.10.28.24314051.](http://paperpile.com/b/rYOPiV/4XV9C)

21.	[Muratore, C. R. *et al.* The familial Alzheimer’s disease APPV717I mutation alters APP processing and Tau expression in iPSC-derived neurons. *Hum Mol Genet* 23, 3523–3536 (2014).](http://paperpile.com/b/rYOPiV/9wY2V)

22.	[Miyashita, A. *et al.* SORL1 is genetically associated with late-onset Alzheimer’s disease in Japanese, Koreans and Caucasians. *PLoS One* 8, e58618 (2013).](http://paperpile.com/b/rYOPiV/D1d0X)

23.	[Rovelet-Lecrux, A. *et al.* APP locus duplication causes autosomal dominant early-onset Alzheimer disease with cerebral amyloid angiopathy. *Nat Genet* 38, 24–26 (2006).](http://paperpile.com/b/rYOPiV/zc4cv)

24.	[Le Guennec, K. *et al.* Deletion of exons 9 and 10 of the Presenilin 1 gene in a patient with Early-onset Alzheimer Disease generates longer amyloid seeds. *Neurobiol Dis* 104, 97–103 (2017).](http://paperpile.com/b/rYOPiV/3qWTd)

25.	[Clarimón, J., Andrés, A. M., Bertranpetit, J. & Comas, D. Comparative analysis of Alu insertion sequences in the APP 5’ flanking region in humans and other primates. *J Mol Evol* 58, 722–731 (2004).](http://paperpile.com/b/rYOPiV/TKMHT)

26.	[Huang, W. *et al.* Genetic risks of Alzheimer’s by APOE and MAPT on cortical morphology in young healthy adults. *Brain Commun* 5, fcad234 (2023).](http://paperpile.com/b/rYOPiV/d6qtm)

27.	[Le Guennec, K. *et al.* 17q21.31 duplication causes prominent tau-related dementia with increased MAPT expression. *Mol Psychiatry* 22, 1119–1125 (2017).](http://paperpile.com/b/rYOPiV/nplNS)

28.	[van Bree, E. J. *et al.* A hidden layer of structural variation in transposable elements reveals potential genetic modifiers in human disease-risk loci. *Genome Res* 32, 656–670 (2022).](http://paperpile.com/b/rYOPiV/s2AFJ)

29.	[Serrano-Pozo, A., Das, S. & Hyman, B. T. APOE and Alzheimer’s disease: advances in genetics, pathophysiology, and therapeutic approaches. *Lancet Neurol* 20, 68–80 (2021).](http://paperpile.com/b/rYOPiV/LIXts)

30.	[De Roeck, A., Van Broeckhoven, C. & Sleegers, K. The role of ABCA7 in Alzheimer’s disease: evidence from genomics, transcriptomics and methylomics. *Acta Neuropathol* 138, 201–220 (2019).](http://paperpile.com/b/rYOPiV/wKBgd)

31.	[Lu, J. *et al.* Association between ABCA1 gene polymorphisms and the therapeutic response to donepezil therapy in Han Chinese patients with Alzheimer’s disease. *Brain Res Bull* 140, 1–4 (2018).](http://paperpile.com/b/rYOPiV/1h8Js)

32.	[De Roeck, A. *et al.* An intronic VNTR affects splicing of ABCA7 and increases risk of Alzheimer’s disease. *Acta Neuropathol* 135, 827–837 (2018).](http://paperpile.com/b/rYOPiV/vVjMa)

33.	[Duchateau, L. *et al.* CSF biomarker analysis of ABCA7 mutation carriers suggests altered APP processing and reduced inflammatory response. *Alzheimers Res Ther* 15, 195 (2023).](http://paperpile.com/b/rYOPiV/dR8xG)

34.	[Gao, Y. *et al.* ZCWPW1 is associated with late-onset Alzheimer’s disease in Han Chinese: a replication study and meta-analyses. *Oncotarget* 7, 20305–20311 (2016).](http://paperpile.com/b/rYOPiV/Hvkat)

35.	[The relationship between four GWAS-identified loci in Alzheimer’s disease and the risk of Parkinson’s disease, amyotrophic lateral sclerosis, and multiple system atrophy. *Neuroscience Letters* 686, 205–210 (2018).](http://paperpile.com/b/rYOPiV/kRkrS)

36.	[Han, S. *et al.* Identification of exon skipping events associated with Alzheimer’s disease in the human hippocampus. *BMC Med Genomics* 12, 13 (2019).](http://paperpile.com/b/rYOPiV/0UgY5)

37.	[Zheng, X. *et al.* Genome-wide copy-number variation study of psychosis in Alzheimer’s disease. *Transl Psychiatry* 5, e574 (2015).](http://paperpile.com/b/rYOPiV/Bdmxw)

38.	[Seripa, D. *et al.* The RELN locus in Alzheimer’s disease. *J. Alzheimers. Dis.* 14, 335–344 (2008).](http://paperpile.com/b/rYOPiV/mpA9)

39.	[Wu, W., Jiang, H., Wang, M. & Zhang, D. Meta-analysis of the association between urokinase-plasminogen activator gene rs2227564 polymorphism and Alzheimer’s disease. *Am J Alzheimers Dis Other Demen* 28, 517–523 (2013).](http://paperpile.com/b/rYOPiV/KVRM8)

40.	[Hao, Y., Li, C., Wang, H. & Ming, C. Effects of copy number variations on longevity in late-onset Alzheimer’s disease patients: insights from a causality network analysis. *Front Aging Neurosci* 15, 1241412 (2023).](http://paperpile.com/b/rYOPiV/80C8W)

41.	[Huang, H. *et al.* The TOMM40 gene rs2075650 polymorphism contributes to Alzheimer’s disease in Caucasian, and Asian populations. *Neurosci Lett* 628, 142–146 (2016).](http://paperpile.com/b/rYOPiV/qEdMn)

42.	[Roses, A. D. *et al.* A TOMM40 variable-length polymorphism predicts the age of late-onset Alzheimer’s disease. *Pharmacogenomics J* 10, 375–384 (2010).](http://paperpile.com/b/rYOPiV/CCY34)

43.	[Larsen, P. A. *et al.* The Alu neurodegeneration hypothesis: A primate-specific mechanism for neuronal transcription noise, mitochondrial dysfunction, and manifestation of neurodegenerative disease. *Alzheimers Dement* 13, 828–838 (2017).](http://paperpile.com/b/rYOPiV/3p5R7)
